# Untargeted metabolomics on first trimester serum implicates metabolic perturbations associated with BMI in development of hypertensive disorders: a discovery study

**DOI:** 10.3389/fnut.2023.1144131

**Published:** 2023-07-17

**Authors:** Yuanyuan Li, Ke Pan, Susan L. McRitchie, Emily W. Harville, Susan C. J. Sumner

**Affiliations:** ^1^Department of Nutrition, Nutrition Research Institute, University of North Carolina at Chapel Hill School of Public Health, Chapel Hill, NC, United States; ^2^Department of Epidemiology, Tulane University School of Public Health and Tropical Medicine, New Orleans, LA, United States

**Keywords:** 1st-trimester, untargeted metabolomics, hypertensive disorders in pregnancy, BMI, metabolic perturbation

## Abstract

**Goal:**

Body mass index (BMI) in early pregnancy is a critical risk factor for hypertensive disorders of pregnancy (HDP). The pathobiology of the interplay between BMI and HDP is not fully understood and represents the focus of this investigation.

**Methods:**

BMI and 1st-trimester serum samples were obtained from the Global Alliance to Prevent Prematurity and Stillbirth repository for 154 women (105 without HDP and 49 with HDP). Metabotyping was conducted using ultra-high-performance liquid-chromatography high-resolution mass spectrometry (UHPLC HR-MS). Multivariable linear regression and logistic models were used to determine metabolites and pathway perturbations associated with BMI in women with and without HDP, and to determine metabolites and pathway perturbations associated with HDP for women in categories of obese, overweight, and normal weight based on the 1st trimester BMI. These outcome-associated signals were identified or annotated by matching against an in-house physical standards library and public database. Pathway analysis was conducted by the Mummichog algorithm in MetaboAnalyst.

**Result:**

Vitamin D3 and lysine metabolism were enriched to associate with BMI for women with and without HDP. Tryptophan metabolism enrichment was associated with HDP in all the BMI categories. Pregnant women who developed HDP showed more metabolic perturbations with BMI (continuous) than those without HDP in their 1st-trimester serum. The HDP-associated pathways for women with normal weight indicated inflammation and immune responses. In contrast, the HDP-associated pathways for women of overweight and obese BMI indicated metabolic syndromes with disorders in glucose, protein, and amino acid, lipid and bile acid metabolism, and oxidative and inflammatory stress.

**Conclusion:**

High first-trimester BMI indicates underlying metabolic syndromes, which play critical roles in HDP development. Vitamin D3 and tryptophan metabolism may be the targets to guide nutritional interventions to mitigate metabolic and inflammatory stress in pregnancy and reduce the onset of HDP.

## Introduction

Maternal obesity is a global public health concern affecting pregnancy wellness, fetal and neonatal outcomes, and other long-term health outcomes for both mothers and their offspring (1–4). The prevalence of obesity globally [Body Mass Index (BMI) ≥30 kg/m^2^] among women has risen from 6 to 15% between 1975 and 2014 (5). In many middle- and high-income countries, up to 50% of women are overweight or obese immediately before or during early pregnancy (3, 6). Obese pregnant mothers are a high risk of developing pregnancy complications, such as gestational diabetes mellitus, hypertensive disorder in pregnancy (HDP), pre-eclampsia (PE), and eclampsia (7, 8). Infants of obese mothers are at increased risk of developing congenital malformations, stillbirth, and macrosomia (9). Obese mothers and their offspring are more likely to develop diabetes and cardiovascular problems in later life (3, 10).

Amongst the pregnancy complications, HDP complicates 6–8% of pregnancies and is one of the leading causes of maternal mortality (11, 12). Gestational hypertension (GH) and PE are two common subtypes of HDP. GH is defined by the new onset of hypertension at ≥20 weeks of gestation, while PE refers to pre-existing hypertension with superimposed proteinuria and/or significant end-organ dysfunction or GH with proteinuria or/and significant end-organ dysfunction (13). It is generally acknowledged that maternal obesity is associated with a significantly increased risk of adverse pregnancy and birth outcomes, including HDP (14, 15). Each 5–7 kg/m^2^ increase in pre-pregnancy BMI is associated with twice the risk of developing PE (15), and obese women have a four times higher risk of developing GH than women of normal weight (16). However, the pathophysiology underlying the correlation between BMI and HDP is not fully understood. Previous research revealed a low prediction rate when using pre-pregnancy or 1st-trimester BMI to predict HDP development (17, 18). Therefore, there is an urgent need to further understand the pathobiological mechanisms of obesity, HDP development, and their interconnection. This approach may reveal biomarker (s) that can better predict HDP outcomes and provide guidance for early intervention to prevent or alleviate HDP.

Targeted and untargeted metabolomics approaches have been used to understand the impact of maternal BMI on metabolic perturbations during pregnancy and the association with birth outcomes or to determine biomarker (s) to predict the onset of HDP and preterm birth (19–24). Leveraging the 1st-trimester serum samples from the Global Alliance to Prevent Prematurity and Stillbirth (GAPPS) repository, we have used high-resolution mass spectrometry untargeted metabolomics to study the causal biology and predictive biomarkers for HDP (including PE and GH) and preterm birth (17). Our earlier studies indicated that metabolic signatures improve the predictive power beyond models only using the conventional risk factors, such as 1st trimester BMI, gravidity, tobacco, and drug usage (17). In addition, metabolic perturbations related to protein/amino acid biosynthesis and metabolism, cortisol biosynthesis, cholesterol and sphingolipid transport, lipoprotein metabolism, and metabolic syndromes were found to be associated with HDP risks. In contrast, the risks of preterm birth were mainly associated with autoimmune responses and depressive disorders.

In this study, we interrogated a published untargeted metabolomics dataset focused on biomarker discovery (17) and reanalyzed the data from different angles to dissect the interaction between 1st trimester BMI and HDP development with the insight of metabolic pathway perturbations. More specifically, we determined metabolic profiles and perturbed pathways in 1st-trimester serum associated with maternal 1st-trimester BMI (continuous) for pregnant women who did and did not develop HDP; and identified the metabolic profile and perturbed pathways in 1st-trimester serum associated with HDP in pregnant women who were stratified into obese, overweight, and healthy weight according to standard BMI cutoffs.

## Methods and materials

### Study population

Serum and clinical metadata for 160 pregnant women, 51 with HDP and 109 without HDP (frequency matched by gravidity), were selected from the Global Alliance to Prevent Prematurity and Stillbirth (GAPPS) repository for women who enrolled between 2011 and 2016. Study participants were enrolled during pregnancy in outpatient clinics from the University of Washington Medical Center, Seattle; Swedish Medical Center, Seattle; and Yakima Valley Memorial Hospital, Yakima, WA. Pregnant women ≥14 years of age were eligible to be enrolled in GAPPS Repository. Women who received narcotics in the previous 12 h, were pregnant with multiples, or were in active labor at enrollment were excluded. Participants were followed up to 10 weeks after delivery. For this study, we excluded 6 participants whose BMI was missing, which resulted in a total of 154 (105 non-HDP participants and 49 HDP participants).

### Clinical definition

Diagnosis of HDP was collected from the medical records directly by GAPPS study personnel (17). HDP cases included cases of gestational hypertension and pre-eclampsia. Gestational hypertension was defined by the new onset of hypertension at ≥20 weeks of gestation; pre-eclampsia was defined by either pre-existing hypertension with superimposed proteinuria and/or significant end-organ dysfunction, or gestational hypertension with proteinuria or/and significant end-organ dysfunction.

### Body mass index and sample collection

BMI (measured by healthcare personnel) and serum specimens from pregnant women were obtained at the 1st prenatal care (PNC) visit during the 1st trimester of pregnancy (6^+1^–13^+6^ weeks). Since the previous study shows that the pre-pregnancy BMI and the first 1^st^ trimester BMI are generally identical regarding the BMI category for most women (25), in this study, we used the adult BMI cutoff criteria (26) from the Center for Disease Control and Prevention to define normal weight (NW, with BMI between 18 and 24.9 kg/m^2^), overweight (OW, with BMI between 25 and 29.9 kg/m^2^) and obese (OB, with BMI ≥30 kg/m^2^). The average gestational week, when BMI and serum specimens were obtained, was at 10.51 ± 1.80 weeks and was not different between the HDP participants (10.64 ± 1.73 weeks) and the non-HDP participants (10.45 ± 1.84 weeks).

### Covariates

The participants’ demographics, health history, and lifestyle information before and during early pregnancy were obtained using the questionnaires designed by the GAPPS repository. The covariates in this study included gravidity, maternal age, the gestational age at the time the BMI was measured, and the blood sample collected, maternal race, and whether participants had smoked more than 100 cigarettes in their lifetime.

### Untargeted metabolomics

Untargeted metabolomics data was acquired on the 1st trimester serum samples collected from 2011 to 2016. Metabolomics data were acquired using a Vanquish UHPLC system coupled with a Q Exactive^™^ HF-X Hybrid Quadrupole-Orbitrap^™^ Mass Spectrometer (UHPLC-HRMS; Thermo Fisher Scientific). Procedures for sample preparation, data acquisition, preprocessing, metabolite identification, and annotation have been published in Harville et al. (17). Briefly, serum (50 μL) was mixed with 400-μL methanol (containing 500 ng/mL L-tryptophan-d5) by vortex at 5,000 rpm for 2 min. Quality control samples (QC pools) were made by pooling 7-μL serum from each study sample. Study samples and QC pools were processed with identical procedures. All samples were centrifuged at 16,000 rcf for 5 min at 4°C. The supernatant (350-uL) was dried and reconstituted with 100 μL water–methanol (95:5, v/v) for data acquisition. Study samples were randomized before sample preparation and acquisition with QC pools interspersed. Metabolites were separated via an HSS T3 C18 column (2.1 × 100 mm, 1.7 μm, Waters Corporation) at 50°C with mobile phases of water (A) and methanol (B), each containing 0.1% formic acid (v/v). The untargeted data were acquired from 70 to 1,050 m/z under the data dependent acquisition mode.

The acquired data was processed by Progenesis QI (version 2.1, Waters Corporation) for peak picking, alignment, and normalization. Signals that were highly varied (RSD > 50%) across QC pools, significantly differed amongst running QC pools batches (ANOVA, with FDR correction *q* < 0.05), or missing in all QC pool samples, were excluded for further analysis.

Metabolite identification/annotation was conducted by Progenesis QI via matching against the in-house experimental standards library (IESL) that was built by acquiring data from over 2,400 compounds under identical conditions to study samples. Signals were also matched to public database, including HMDB, NIST, and METLIN. The evidence supporting each identification or annotation was labeled with the Ontology level (e.g., OL1-PDd), which were shown and further explained in Supplementary Tables S1, S2.

### Statistical analysis

All statistical analyses were performed using R (version 3.6.1) within the R Studio (version 1.3.1093) platform. For normally distributed continuous variables, we calculated the mean and standard deviation. For continuous variables not normally distributed, we calculated median, 25th percentile, and 75th percentile. The student’s *t*-test or analysis of variance (ANOVA) test was used for parametric data depending on the number of comparison groups, while the Wilcoxon rank-sum test or Kruskal-Wallis test was used for nonparametric data. The number of participants and percentage for categorical variables and compared across groups using the Chi-square test.

**Figure 1:**
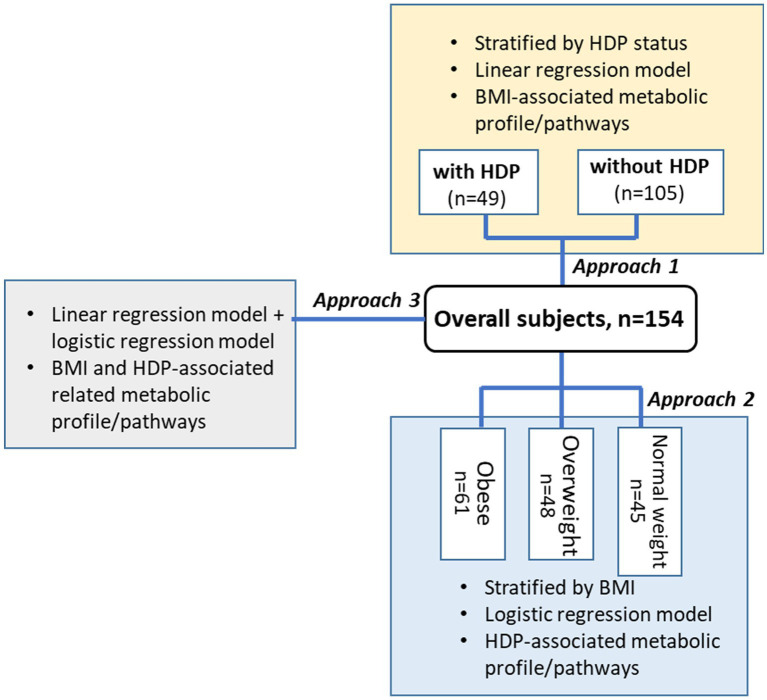
Study design and data analysis approach. Samples and covariates (BMI, cigarette consumption, etc.) were collected in 1st trimester during the first clinical visit. None of the participants were diagnosed with HDP on their first visit.

We used 3 modeling approaches to determine signals associated with BMI and HPD (Figure 1). For approach 1, we used linear regression models to determine the BMI (continuous) associated signals in women who developed HDP (*n* = 49) and who did not develop HDP (*n* = 105), respectively. Multiple linear regression models were used to adjust for maternal age, whether they smoked more than 100 cigarettes in their lifetime and gestational week when serum samples and BMI were obtained. The False Discovery Rate (FDR) adjustment was used to correct for multiple testing. A two-sided FDR corrected *p*-value (or *q*-value) of <0.05 was considered significant.

For approach 2, we first stratified the 154 participants into 3 BMI categories: NW (BMI < 25 kg/m^2^, *n* = 45); OW (25 kg/m^2^ ≤ BMI < 30 kg/m2, *n* = 48), and OB (BMI > 30 kg/m^2^, *n* = 61). Within each BMI subgroup, we used logistic regression models to determine signals associated with HDP by adjusting gravidity (frequency-matching variable) and maternal age. Because of the small sample size, the original nominal *p*-value < 0.05 (without FDR adjustment) was considered significant for this discovery investigation.

For approach 3, we first used linear regression models to determine signals significantly associated with BMI among all 154 participants (105 without HDP, 49 with HDP), adjusting for maternal age, whether they smoked more than 100 cigarettes in their lifetime, and gestational week when serum samples and BMI were obtained. Within the list of signals associated with BMI, we used logistic regression models adjusting for gravidity (frequency-matching variable) to determine signals associated with BMI and HDP. A two-sided FDR corrected *p*-value (or *q*-value) of <0.05 was considered significant.

Multivariable linear or logistic regression models adjusting for potential confounders were used to estimate the adjusted effect sizes. Covariates associated with both the predictor and the outcome variable, but not on the causal pathway, in each model were considered potential confounders. All peaks meeting the significance level were considered in the analysis. A complete case analysis approach was used. Covariate missing data were minimal: maternal age (1%) and whether they smoked more than 100 cigarettes in their lifetime (4%).

### Pathway enrichment

Pathway analysis was conducted using MetaboAnalyst 5.0 via the Functional Analysis (MS Peaks module), built on the Mummichog algorithm (27–30). All normalized signals (m/z) that remained after preprocessing and quality control were uploaded to the pathway analysis tool, together with the *p*-value calculated by the regression models (without adjusting covariates and FDR correction) to indicate the association between signal and outcomes (e.g., BMI and/or HDP). An original nominal *p*-value cut-off of 0.05 was used to determine the size of the permutation group that the algorithm used for selecting significant signals to match all possible metabolites. A 3-ppm mass accuracy tolerance was used in signal annotations to identify candidate pathways. All possible metabolites matched by m/z were searched in the *Homo sapiens* (human) [MFN] pathway library.

To further understand the direction of change impacted by BMI and HDP status for specific pathways, we verified the enriched empirical annotations made via the Mummichog algorithm by matching the corresponding signals against our in-house physical standard library or public bases (NIST, HMDB). We then used box plots to demonstrate the up- and down of these key metabolites in the enriched pathway according to different pairwise comparisons using peak intensity.

## Results

### Subject characteristics data for women with different BMI and HDP status

The average maternal age for all 154 participants was 29.7 ± 5.3 years old, and the average gestational age for BMI measurement and serum samples collection was 10.5 ± 1.8 weeks. There was no significant difference between participants with and without HDP in maternal and gestational age when BMI and serum samples were collected (Table 1). About 71% of the participants were white, and there was no significant difference in demographic composition between groups with- and without HDP. The overall percentage of participants who smoked more than 100 cigarettes in their lifetime was 22.3%, and the percentage was significantly higher in the HDP group (33.3%) than in the group without HDP (17%) (*p* = 0.03). The mean gestational age of delivery was 39.3 weeks for all participants, while participants who developed HDP delivered earlier on average compared to the non-HDP participants [median (q1–q3): 38.6 (37.9–40.0) vs. 39.3 (38.9–40.3), *p* < 0.001].

**Table 1:** Demographic, medical, and lifestyle characteristics of participants.

	Overall (*n* = 154)	Non-HDP (*n* = 105)	HDP (*n* = 49)	*p* value	Normal weight (*n* = 45)	Overweight (*n* = 48)	Obese (*n* = 61)	*p* value
Maternal age (mean ± SD)	29.72 ± 5.25	29.85 ± 5.14	29.44 ± 5.52	0.67^a^	30.40 ± 4.95	29.17 ± 5.25	29.64 ± 5.49	0.64^b^
BMI at first prenatal visit (median, q1–q3)	28.46, 24.46–35.67	27.27, 23.76–31.47	34.29, 28.12–40.39	<0.001^c^	22.98, 22.11–24.96	27.68, 26.44–28.72	39.67, 34.29–43	<0.001^d^
Gestational age when BMI and serum sample were collected (mean ± SD)	10.51 ± 1.80	10.45 ± 1.84	10.64 ± 1.73	0.52^a^	10.51 ± 1.88	10.44 ± 1.74	10.56 ± 1.82	0.83*
Gravidity [*n* (%)]				0.74^e^				0.003^e^
1	35 (22.73)	24 (22.86)	11 (22.45)		17 (37.8)	12 (25)	6 (9.8)	
2	44 (28.57)	29 (27.62)	15 (30.61)		11 (24.4)	16 (33.3)	17 (27.9)	
3	42 (27.27)	27 (25.71)	15 (30.61)		12 (26.7)	14 (29.2)	16 (26.2)	
>3	33 (21.43)	25 (23.81)	8 (16.33)		5 (11.1)	6 (23.5)	22 (36.1)	
Race				0.90^e^				0.32^e^
White	109 (70.8)	74 (70.5)	35 (71.4)		34 (75.6)	36 (75)	39 (63.9)	
Non-white	45 (29.2)	31 (29.5)	14 (28.6)		11 (24.4)	12 (25)	22 (36.1)	
Pre-pregnancy obesity (medical records) [*n* (%)]	56 (37.0)	30 (29.4)	26 (53.1)	0.01^e^	1 (2.3)	4 (8.5)	51 (83.6)	<0.001^e^
Gestational age at delivery (median, q1–q3)	39.29, 38.43–40.14	39.29, 38.86–40.29	38.57, 37.85–40.00	<0.001^c^	39.43, 38.86–40.29	39.36, 38.68–40.07	39.00, 38.14–40.00	0.07 ^d^
Smoked more than 100 cigarettes (about 5 packs) in your lifetime [*n* (%)]	33 (22.3)	17 (17.0)	16 (33.3)	0.03^e^	7 (16.3)	12 (26.7)	14 (23.3)	0.49^e^

SD, standard deviation; q1, 25th percentile; q3, 75th percentile.

a Student’s *t*-test.

b Analysis of variance (ANOVA) test.

c Wilcoxon rank sum test.

d Kruskal-Wallis test.

e Chi-square test.

Participants who developed HDP showed significantly higher 1st PNC BMI than those without HDP [median (q1–q3): 34.29 (28.1–40.4) vs. 27.27 (23.8–31.5), *p* < 0.001] (Table 1). Among different BMI categories classified according to the 1st PNC measurements, 6 out of 45 (13%) women in NW developed HDP, 14 out of 48 (29%) women in OW developed HDP, and 29 out of 61 (48%) participants in OB developed HDP.

### BMI-associated metabolic profiles and pathways in women with and without HDP

**Figure 2:**
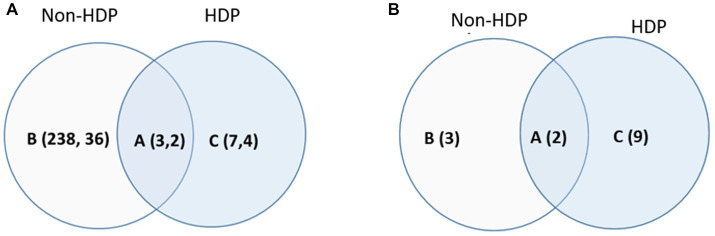
Venn diagram for the BMI-associated signal/metabolites **(A)** and the enriched pathways **(B)** in women with- and without HDP. **(A)** The BMI-associated signals (the first number) and identified/annotated metabolites (the second number) determined in women (A) with- and without HDP; (B) without HDP only; (C) with HDP only. The association was determined by the linear regression models (*p* < 0.05 and corrected by FDR) adjusting for maternal age, whether they smoked more than 100 cigarettes in their lifetime, and gestational week when serum samples and BMI were obtained. Signals and the identified (or annotated) metabolites corresponding to **(A)** were summarized in Supplementary Table S1. **(B)** The enriched BMI-associated pathway for women (A) with and without HDP, (B) without HDP only, and (C) with HDP only by Metaboanalyst 5.0 (Funtional Analysis MS peak module). The list of pathways corresponding to **(B)** were summarized in Table 2i. No covariates was adjusted for data used for pathway enrichment.

We stratified the 154 participants into 2 groups: with- (*n* = 49) and without (*n* = 105) HDP, and then used linear regression models to determine metabolic profiles and pathways that are associated with BMI (continued variance). After adjusting for confounding factors, including maternal age, tobacco usage (lifetime smoking ≥100 cigarettes), and the gestational week of the blood draw and BMI measurement, 241 signals were determined to associate with BMI in women without HDP, with 38/241 signals being identified or annotated via matching against the in-house library and public database (Figure 2A; Supplementary Table S1). In contrast, 10 signals were associated with BMI in women with HDP, with 6/10 signals identified or annotated. Three BMI-associated signals overlapped between participants with and without HDP (Figure 2A), and 2/3 were annotated as steroids/hormones and vitamin D3-related metabolites (Supplementary Table S1).

Pathway enrichment was performed using all 3,300 normalized signals, regardless of being identified/annotated, together with the *p*-value that indicated the statistical strength of association with BMI. As shown in Figure 2B and Table 2i, 5 BMI-associated pathways were enriched for women without HDP, while 11 BMI-associated pathways were enriched for women with HDP. The shared BMI-associated pathways for women with and without HDP were vitamin D3 and lysine metabolism. Three BMI-associated pathways unique to women without HDP related to energy balance and mitochondrion function, including the TCA cycle, vitamin B5-CoA biosynthesis from pantothenate, and ubiquinone biosynthesis. Comparatively, more perturbed pathways were found to associate with BMI in women with HDP, involving lipid and fatty acid metabolism (carnitine shuttle, glycerophospholipids metabolism), one-carbon metabolism (methionine and cysteine metabolism), chronic-low grade inflammation (tryptophan and tyrosine metabolism, glutathione metabolism), and xenobiotics (cytochrome P450) (Table 2i).

**Table 2:** Enriched metabolic pathways associated with different outcomes.

Region codes^a^	Enriched pathways	Outcome	Number of the empirical compound perturbed in the enriched pathway^b^	Gamma *p* value^c^
**(i) Enriched pathways associated with BMI in women with- and without HDP**				
A (2)	Vitamin D3 (cholecalciferol) metabolism*	Non-HDP	6/9/16	0.02
		HDP	4/9/16	<0.01
	Lysine metabolism*	Non-HDP	8/18/52	0.05
		HDP	3/18/52	0.04
B (3)	TCA cycle*	Non-HDP	5/6/31	0.02
	Vitamin B5 - CoA biosynthesis from pantothenate	Non-HDP	3/3/12	0.03
	Ubiquinone Biosynthesis*	Non-HDP	4/6/10	0.03
C (9)	Tryptophan metabolism*	HDP	12/58/94	0.01
	Tyrosine metabolism*	HDP	14/76/160	0.01
	Glycerophospholipid metabolism*	HDP	6/27/156	0.01
	Xenobiotics metabolism*	HDP	5/28/110	0.02
	Glutathione Metabolism	HDP	2/6/19	0.02
	Carnitine shuttle	HDP	3/16/72	0.03
	Ascorbate (Vitamin C) and Aldarate Metabolism	HDP	2/9/29	0.04
	Methionine and cysteine metabolism	HDP	3/18/94	0.04
	Drug metabolism—cytochrome P450*	HDP	4/28/53	0.05
**(ii) Enriched metabolic pathways associated with HDP in pregnant women stratified by BMI category**				
A (1)	Tryptophan metabolism*	Normal	2/58/94	0.05
		Overweight	4/58/94	0.00
		Obese	5/58/94	0.04
B (1)	Prostaglandin formation from arachidonate	Normal	2/40/78	0.03
		Overweight	2/41/78	0.01
D (6)	Glycerophospholipids metabolism*	Overweight	3/27/156	0.00
		Obese	5/27/156	0.00
	Ubiquinone Biosynthesis*	Overweight	2/6/10	0.00
		Obese	2/6/10	0.01
	Urea cycle/amino group metabolism	Overweight	3/32/85	0.00
		Obese	3/32/85	0.04
	Lysine metabolism*	Overweight	2/18/52	0.01
		Obese	2/18/52	0.05
	Androgen and estrogen biosynthesis and metabolism	Overweight	3/48/95	0.01
		Obese	4/48/95	0.05
	Tyrosine metabolism*	Overweight	3/79/160	0.01
		Obese	6/76/160	0.05
E (3)	Fatty acid activation	Normal	2/17/74	0.01
	Leukotriene metabolism	Normal	2/26/92	0.02
	Arachidonic acid metabolism	Normal	2/58/94	0.05
*F* (6)	Histidine metabolism	Overweight	3/9/33	0.00
	Methionine and cysteine metabolism	Overweight	3/18/94	0.00
	Vitamin B12 (cyanocobalamin) metabolism	Overweight	2/2/9	0.00
	Glycine, serine, alanine and threonine metabolism	Overweight	3/27/88	0.00
	Vitamin B3 (nicotinate and nicotinamide) metabolism	Overweight	2/12/28	0.01
	Aspartate and asparagine metabolism	Overweight	2/47/114	0.01
G (10)	Vitamin D3 (cholecalciferol) metabolism*	Obese	3/9/16	0.00
	Pyrimidine metabolism	Obese	3/15/70	0.01
	TCA cycle*	Obese	2/6/31	0.01
	Xenobiotics metabolism*	Obese	4/28/34	0.01
	Pyranic acid peroxisome oxidation	Obese	2/8/34	0.01
	Drug metabolism - cytochrome P450*	Obese	3/28/53	0.03
	Butanoate metabolism	Obese	2/14/34	0.03
	Glycosphingolipid metabolism	Obese	2/14/67	0.03
	*De novo* fatty acid biosynthesis	Obese	2/15/106	0.03
	Bile acid biosynthesis	Obese	3/34/82	0.05

**Figure 3:**
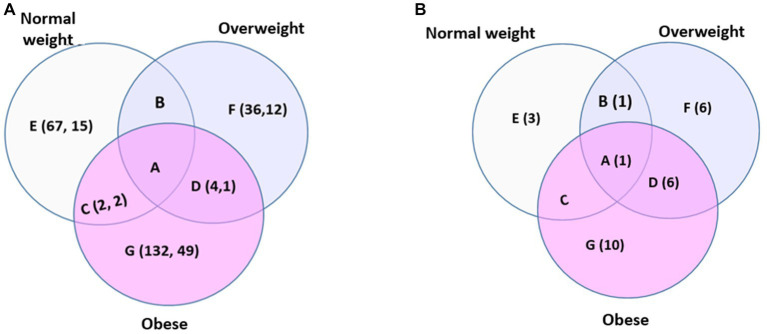
Venn diagram for the HDP-associated signal/metabolites **(A)** and pathways **(B)** in women within different BMI categories. **(A)** The HDP-associated signals (the first number) and metabolites (the second number) determined in women of (A) all three BMI categories, (B) overweight and normal weight, (C) normal weight and obese, (D) overweight and obese, (E) normal weight only, (F) overweight only, and (G) obese only. The association was determined by logistic regression models (*p* < 0.05) adjusting for gravidity and maternal age, whether they smoked more than 100 cigarettes in their lifetime, and gestational week when serum samples and BMI were obtained. Signals and the identified (or annotated) metabolites corresponding to **(A)** were summarized in Supplementary Table S2. **(B)** The enriched HDP-associated pathway for women of (A) all three BMI categories, (B) overweight and normal weight, (C) normal weight and obese, (D) overweight and obese, (E) normal weight only, (F) overweight only, and (G) obese only. The list of pathways corresponding to **(B)** was summarized in Table 2ii. No covariates were adjusted for data used for pathway enrichment.

(i) The BMI-associated metabolic pathways in women with- and without HDP and (ii) The HDP-associated metabolic pathways in women of healthy weight, overweight and obese were enriched by MetaboAnalyst 5.0 via the functional analysis (MS peaks) module.

a. Regional codes: for (i), capital letters (A, B, and C) and numbers correspond to the Venn diagram in Figure 2B; for (ii), capital letters (A–G) and numbers correspond to the Venn diagram in Figure 3B.

b. Number of the empirical compound perturbed in the enriched pathway: x/y/z, “x” indicates the total number of empirical compounds in the pathway, “y” indicates the number of empirical compounds annotated in the untargeted metabolomics dataset, “z” indicates the number of empirical compounds significantly perturbed (*p* < 0.05) with HDP.

c. Gamma *p*-value, the Gamma adjusted *p*-value indicates the significance of the pathway enrichment. A lower *p*-value indicates a greater significance in the enrichment. The cut-off for pathway significance is *p* < 0.05.

* Overlapped Pathways between (i) Enriched pathways associated with BMI in women with- and without HDP and (ii) Enriched metabolic pathways associated with HDP in pregnant women stratified by BMI category.

### HDP-associated metabolic profiles and pathways in women within different BMI categories: NW, OW, and OB

We stratified the 154 participants into 3 BMI categories: NW (*n* = 45, BMI < 25 kg/m^2^), OW (*n* = 48, 25 kg/m^2^ ≤ BMI < 30 kg/m^2^), and OB (61 subjects, BMI > 30 kg/m^2^) and used logistic regression models to determine the HDP-associated metabolic profiles and pathways in each BMI category.

The number of signals/metabolites associated with HDP was 69, 40, and 138 for the NW, OW, and OB sub-group, respectively, after covariate adjustment (Figure 3A; Supplementary Table S2). The HDP-associated metabolites/signals in each of the BMI sub-group seemed quite unique, as no shared signal/metabolite was determined across the three categories, only 2 signals/metabolites overlapped between the NW and OB, and only 4 signals/metabolites were overlapped between OW and OB (Figure 3A; Supplementary Table S2). The identified or annotated HDP-associated metabolites in the NW sub-group include tryptophan derivatives (e.g., 5-hydroxyindoleacetic acid, OL1), polyphenols derivatives (e.g., 2,6-Di-tert-butyl-4-hydroxymethylphenol, PDa), and arachidonic acid metabolites. In contrast, the HDP-associated metabolites in the OB sub-groups include nucleosides (e.g., cytidine, OL1), tryptophan derivatives (e.g., acetylserotonin, OL2b, indoleacetic acid, OL2b, 3,4-dihydroxyphenylacetic acid, OL2b), cholic acid metabolites (e.g., dehydrolithocholic acid, OL2b, glycylcholic acid, OL2b), hormones (e.g., 4-androstene-3,17-dione, PDa, and 5-androstene-3.beta.-ol-17-one, PDa), and polyphenol derivatives (e.g., 3,4,5-trimethoxybenzaldehyde, OL1). Compared to the NW and OB subgroups, relatively fewer signals/metabolites are associated with HDP in the OW subgroup, and many are lipid and fatty acid-related compounds.

The number of pathways associated with HDP was 5, 14, and 17 for the NW, OW, and OB, respectively (Figure 3B; Table 4). Tryptophan metabolism is associated with HDP for all 3 BMI subgroups. Six pathways were associated with HDP in both the OW and OB sub-groups (Table 2ii), mainly involving nutrients metabolism and energy balance (e.g., glycerophospholipids metabolism, ubiquinone biosynthesis, urea cycle/amino group metabolism, lysine- and tyrosine metabolism) and sex hormone hemostasis (androgen and estrogen biosynthesis and metabolism). There was no overlap of HDP-associated pathways between the OB and NW subgroup, and the only pathway shared between NW and OW subgroups is related to inflammation and immune response (prostaglandin formation from arachidonate). The HDP-associated pathways unique to the NW subgroup were all about arachidonic metabolism and inflammation (e.g., fatty acid activation, leukotriene metabolism, and arachidonic acid metabolism). In contrast, the pathways unique to OW or OB subgroups were mainly involved in nutrient metabolisms, such as vitamins, sugar, bile acids, lipids, amino acids, and proteins (Table 2ii).

### BMI and HDP-associated signals/metabolites

We used linear and logistical regression models to determine signals/metabolites simultaneously associated with BMI and HDP based on the overall 154 participants. After adjusting for covariates, three signals/metabolites remained in the model. One of the three signals was identified as cytidine (OL1), and the other two were annotated as metabolites from glycerophospholipids and vitamin D3 metabolism (Table 3). Since there were only a few signals (*n* < 10) that showed significant association (*p* < 0.05) with the outcomes using this approach, regardless of whether under covariates adjustment or FDR correction or not, we did not receive meaningful pathway enrichment results.

**Table 3:** The determined signals/metabolites associated with BMI and HDP by linear regression modeling (*n* = 154).

Signal	Compound ID	Annotation/identification	Ontology	Odds ratio*	95%CI	FDR *p* value^#^
0.94_244.0926 m/z	6175	Cytidine	OL_1	2.22	1.41–3.50	0.032
14.51_631.3452 m/z	1114770-15-8	1-(9Z-Octadecenoyl)-sn-glycero-3-phospho-(1′-myo-inositol)	PDc	2.18	1.43–3.32	0.026
13.56_402.3130n	131769837	24-oxo-1alpha,25-dihydroxyvitamin D3^++^	PDd	2.34	1.51–3.62	0.025

* Linear regression models adjusting for maternal age, whether smoked more than 100 cigarettes in their lifetime, and gestational week when serum samples and BMI were obtained were used to identify signals that were associated with BMI; Logistic regression models further adjusting for gravidity (frequency-matching variable) were used to identify BMI-associated signals that were associated with HDP.

# False Discovery Rate (FDR) corrected *p*-value.

++ Annotation is made by matching against the public base with MS (PDd) and supported by the pathway enrichment results.

### Vitamin D3 and tryptophan metabolism

**Figure 4:**
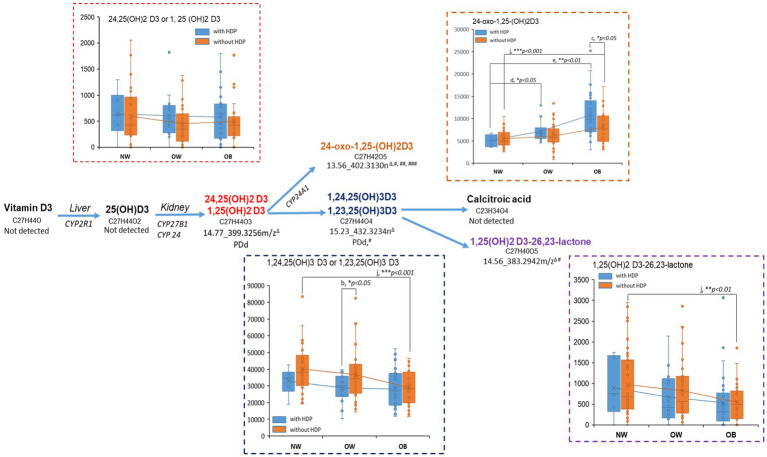
Pertubation of metabolites on vitamin D pathway regarding BMI and HDP status. Metabolites in the enriched vitamin D3 pathway were first annotated by the Mummichog algorism in Metabanalyst via the empirical compound library and then verified by matching against the public database. PDd indicated the annotation was supported by the exact mass match in the public database. The current untargeted approach may not unambiguously identify the structural isomers because of lacking experimental spectra and chromatographic information in the in-house library. #metabolite determined to associate with BMI in the non-HDP and/or HDP group (Supplementary Table S1); ## metabolite determined to associate with HDP in at least one of the BMI categories (Supplementary Table S2); ### metabolite determined to associate with HDP and BMI by regression modeling (Table 3). The box plots of the annotated metabolites were based on the relative peak intensity of the corresponding signal (^Δ^RT_exact mass) and the classification with BMI categories (NW, normal weight; OW, overweight; OB, obese) and the HDP status (with-HDP, without HDP). The pairwise comparison between BMI category or between HDP status was conducted by student *t*-test or Wilcoxon Rank-Sum Test (if the sample size in one of the groups is <10). Pairwise comparisons: (a) HDP vs. without-HDP in NW; (b) with-HDP vs. without-HDP in OW; (c) with-HDP vs. without-HDP in OB; (d) OW vs. NW in with-HDP; (e) OB vs. NW in with-HDP; (f) OW vs. NW in without-HDP; (g) OB vs. NW in without-HDP. Comparisons with significant changes were labled in the box plots (*p < 0.05, **p < 0.01, ***p < 0.001).

In the enriched vitamin D3 metabolism (Figure 4), dihydroxylated [1,25(OH)2D3], tri-hydroxylated [1,24,25(OH)3D3], and the two oxidative metabolites [24-oxo-1,25 (OH)2D3, 1,25-(OH)2D3-26,23-lactone] were empirically annotated by the Mummichog algorithm, and the dihydroxylated- and tri-hydroxylated D3 were verified via matching the exact mass with public base. We observed a consistent trend of decrease in the vitamin D3 metabolites with BMI increment, especially for women without HDP (Figure 4), except for the C-24 oxidation metabolite 24-oxo-1,25 (OH)2D3, which was increased with the BMI increment, especially for the women with HDP. Within the category of OB, we observed a significant increase of 24-oxo-1,25 (OH)2D3 levels in women with HDP compared to those without HDP (*p* < 0.05).

**Figure 5:**
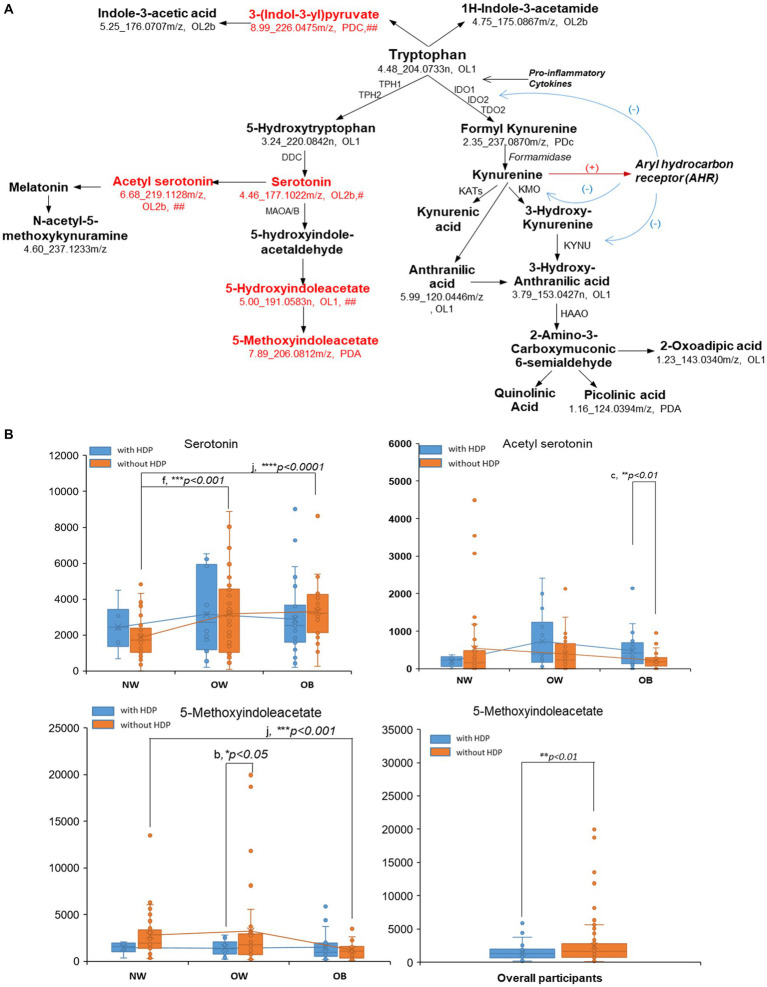
Pertubation of metbaolites on tryptophan pathway regarding BMI and HDP status. Metabolites in the tryptophan pathway **(A)** were first annotated by the Mummichog algorism in Metabanalyst via the empirical compound library, and then verified by matching against the in-house physical standard library (OL1-OL2b) and public database (PDa-PDd). OL1, matching with In-house physical standard library (IPSL) via retention time (RT), exact mass (MS), and tandem mass similarity (MS/MS); OL2a, matching with IPSL via MS and RT; and OL2b, the annotation for the isomer or derivatives of the compound, based on matching with IPSL via MS and MS/MS. PDa, annotation based on public database via MS and experimental MS/MS (could be the listed compound, or the isomer or derivatives of the listed compound); PDc, annotation based on public database via exact mass and isotopic similarity. #metabolite determined to associate with BMI in the non-HDP and/or HDP group (Supplementary Table S1); ## metabolite determined to associate with HDP in at least one of the BMI categories (Supplementary Table S2); ### metabolite determined to associate with HDP and BMI by regression modeling (Table 3). The box plots **(B)** of the identified/annotated metabolites were based on the relative peak intensity of the corresponding signal (RT_exact mass) and the classification with BMI categories (NW, normal weight; OW, overweight; OB, obese) and the HDP status (blue: with-HDP; orange: without HDP). The pairwise comparison was conducted by student *t*-test or Wilcoxon Rank-Sum Test (if the sample size in one of the group is <10). Pairwise comparisons: (a) HDP vs. without-HDP in NW; (b) with-HDP vs. without-HDP in OW; (c), with-HDP vs. without-HDP in OB; (d) OW vs. NW in with-HDP; (e) OB vs. NW in with-HDP; (f) OW vs. NW in without-HDP; (G) OB vs. NW in without-HDP. Comparisons with significant changes were labled in the box plots (*p < 0.05, **p < 0.01, ***p < 0.001). **(B)** only presented the box plots for metabolites for serotonin, acetyl serotonin, and 5-methoxyindoleacetate. Box plots for other metabolites were shown in Supplementary Figure S1.

As shown in Figure 5A, the majority of the empirically annotated tryptophan metabolites in the enriched pathway were verified by the in-house library within OL1 (RT, MS, MS/MS) or OL2b (MS, MS/MS) or by public databases within PDA (MS, MS/MS) or PDc (MS). Metabolites in the tryptophan-serotonin pathways, including serotonin, acetyl serotonin, 5-hydroxyindoleacetate, and 5-methoxyindoleacetate (5-MIAA), were found to be significantly varied with the BMI or HDP outcomes (Supplementary Table S2; Figure 5B; Supplementary Figure S1). Serotonin and aceylserotonin showed a trend of increase with BMI, and aceylserotonin was significantly increased in obese women with HDP compared to obese women without HDP (*p* < 0.05) (Figure 5B). In contrast, 5-methoxyindoleacetate (5-MIAA) presented an opposite trend compared to serotonin and acetyl serotonin and decreased with the increment of BMI, especially for women without HDP (*p* < 0.001). This metabolite was significantly decreased in women with HDP vs. without HDP in the OW (*p* < 0.05) and overall participants (*p* < 0.01) (Figure 5B).

## Discussion

HDP is one of the leading contributors to maternal mortality. However, the pathophysiologic mechanism is not fully understood. Although early/pre-pregnancy overweight/obesity is recognized as one of the most important risk factors for HDP, it is unclear how BMI increase impacts metabolic perturbations and contributes to HDP development. Leveraging on the untargeted metabolomics data acquired for the first-trimester serum from the GAPPS cohort (17), we used multiple regression models to understand the metabolic perturbations involved with BMI and HDP from different perspectives. The interplay between metabolic syndromes and BMI is strongly associated with HDP development. In addition, systemic inflammation and immune responses contributed to HDP risk for women of NW.

Mothers in the OW and OB categories during early or pre-pregnancy are at higher risk of developing metabolic syndromes, a cluster of disorders encompassing metabolic, vascular, and inflammatory functions (21, 31–33). We found 1st trimester pathway perturbations determined in women who developed HDP at a later stage all pointed to chronic inflammation (tryptophan metabolism), oxidative stress (glutathione metabolism), and interruptions in fatty acid/lipid metabolism (glycerophospholipids metabolism, carnitine shuttle), protein/amino acid biosynthesis and metabolism (lysine, methionine, cysteine, tyrosine metabolism), and vitamin metabolism (vitamin D3 and C), as well as xenobiotics and drug metabolism (cytochrome P450) (Figure 2; Table 2). These enriched BMI-associated pathways for HDP mothers greatly overlapped with the enriched HDP-associated pathways for the OW and OB mothers (Table 2). These findings suggest that high early pregnancy BMI may exacerbate the underlying metabolic syndromes, the key contributors to pregnancy complications, including HDP and gestational diabetes (33, 34). Moreover, environmental exposures and a sedentary lifestyle, including smoking and poor nutrition, may also contribute to the risks of metabolic syndrome and HDP (35–38).

Although the early pregnancy BMI is associated with the risk of HDP, some OW and OB women did not develop HDP, while some NW women developed HDP in late pregnancy. Women without HDP showed less metabolic perturbation associated with BMI in their 1st trimester than those with HDP (Table 2), indicating that individual metabolic wellness, which is impacted by factors including but not limited to inherited genetics, lifestyle, disease history, and maternal exposures, may play important roles in HDP development in addition to the BMI (39–41). The HDP-associated pathways for NW women are all related to inflammation and immune responses (Figure 3; Table 4), consistent with the established mechanism that excessive systemic inflammation and oxidative stress during pregnancy cause endothelial dysfunction and lead to the development of hypertension, especially preeclampsia (42–45). Our findings indicate that in addition to monitoring the pre-or early pregnancy BMI, metabolic biomarker(s) are urgently needed to improve the predictive accuracy of diagnosing HDP in the early stage.

Extensive human and animal research has demonstrated the relationship between vitamin D3 metabolism interruption with overweight/obesity, insulin resistance, diabetes, cardiovascular events, and pregnancy complications (46–48). We observed consistent trends of decreased bioactive vitamin D3 forms, including 1,25(OH)2D3, 1,24,25(OH)3D3, and 1,25-(OH)2D3-26,23-lactone, with the BMI increase, especially for women with HDP. The low vitamin D3 (mainly the bioactive dihydroxylated form) has been revealed to associate with increased renal renin and angiotensin II production, consequently elevating blood pressure or developing essential hypertension (49). It is worth mentioning that one of the oxidative metabolites, 24-oxo-1,25-(OH)2D3, was increased with BMI and was significantly increased in obese women with HDP compared to obese women without DHP (*p* < 0.05). The increase of 24-oxo-1,25-(OH)2D3 might be related to the overexpression of CYP24A1, a cytochrome P450 enzyme that catalyzes the degradation of active vitamin D3 forms into inactive oxidative products (50). The overexpression of CYP24A1 has been revealed to be involved in developing chronic kidney diseases and might be responsible for decreasing vitamin D active forms in circulation (50). We found 24-oxo-1,25-(OH)2D3 was strongly associated with BMI and HDP simultaneously in the regression model after FDR correction and co-variance adjustment (Table 3), indicating that this D3 metabolite could be used to predict the development of HDP, especially for obese women. On the other hand, interventions targeting inhibiting CYP24A1 and maintaining the active forms of vitamin D3 in circulation might be a solution to alleviate hypertensive pregnancy disorders (51).

Our result indicated tryptophan metabolism was associated with HDP in all BMI categories, and the perturbations of metabolites regarding HDP or BMI were mainly concentrated in the tryptophan-serotonin pathway (Supplementary Tables S1, S2; Figure 5; Supplementary Figure S1). We found increases in serotonin and aceylserotonin with BMI increment and HDP development, consistent with findings that activation of tryptophan degradation and the increase of serotonin levels in plasma and placental was related to the risk of HDP, especially for PE: serotonin and tryptophan metabolites triggered immune responses, including vascular hyporeactivity, excess platelet aggregation, and pro-inflammatory processes (52–54). One serotonin metabolite, 5-methoxyindoleacetate (5-MIAA), was decreased with the BMI increase and presented a negative correlation with the HDP risk (Figure 5B). 5-MIAA is a further metabolized product of 5-hydroxy-indole acetate catalyzed via methyltransferase (55). The methoxyindoles, including 5-MIAA, have been revealed as COX inhibitors and the PPARγ activator and are associated with the anti-inflammatory and anti-oxidation process (55, 56). Interventions targeting tryptophan metabolism, either through reducing the overall degradation of tryptophan-serotonin or supplementing the anti-inflammatory and anti-oxidative methoxyindoles, may help alleviate inflammation and adverse immune response for women with HDP risk.

The main strength of this study includes that we took a global approach to interrogate the untargeted data from 3 different angles to understand the relationship and biological insights between early pregnancy BMI and the onset of HDP. Instead of focusing on a small set of the named metabolites, we used a signal-to-pathway approach to enrich the metabolic pathways perturbed regarding BMI and HDP. Then we focused on the most relevant pathways (vitamin D and tryptophan metabolism) to reveal the up-and-down of the key metabolites within different phenotypic groups. We acknowledged several limitations in this study. One is the sample size, especially for women who developed HDP in NW, which limited the power to demonstrate the significant association between metabolites and outcomes. Another limitation was lacking paired longitudinal samples when the pregnancy complications were diagnosed. While early-pregnancy samples are valuable for revealing risk factors early on and predicting the outcomes, analyzing paired biospecimens at disease onset would help us understand the pathobiology of HDP. In addition, due to lack of available data, we missed information on covariates, such as diet, exercise, and drug use. These factors could contribute to BMI, with associated metabolites being downstream via the same pathway, or could cause residual confounding. Moreover, many signals important to the outcomes are still kept unidentified or unannotated, because of the limitation in the available compound library and current data mining technologies. Even so, we have listed these signals with unique physic-chemical features (e.g., exact mass and retention time) and shared the original raw data in the metabolomics data repository, which allows data re-analysis and re-visit in the future to unlock these unknown compounds. Finally, a quantitative targeted approach is needed to verify the concentrations of the vitamin D and tryptophan metabolites and their relationships with the outcomes, especially in another cohort.

## Conclusion

High first-trimester BMI is an indicator of underlying metabolism, which plays critical roles in HDP development. Many other conditions, including an individual’s inherited genetics, nutrition, environmental exposures, metabolic syndrome, autoimmune response, and chronic inflammation level, also contribute to HDP development. If confirmed, our results suggest that interventions targeting vitamin D3 and tryptophan pathways might attenuate metabolic syndromes and chronic inflammation, preventing and reducing HDP from early pregnancy. Future studies utilizing a quantitative targeted approach are needed to validate the current findings.

## Data availability statement

Publicly available datasets were analyzed in this study. This data can be found at: This data is available at the NIH Common Fund’s National Metabolomics Data Repository (NMDR) website, the Metabolomics Workbench (http://dx.doi.org/10.21228/M84M8C).

## Author contributions

YL and KP conceived study, data modeling, identification and annotations of signals, pathway analysis, manuscript preparation, prepared figures, tables, and Supplementary material. SM statistical analysis of subject characteristic data and metabolomics data. EH conceived study, supervised data modeling, and manuscript revision. SS conceived study, supervised metabolomics data analysis, signal identification and annotation, organized the manuscript, and interpreted results. All authors contributed to the article and approved the submitted version.

## Funding

This work was supported by the National Institute of Environmental Health Sciences (NIEHS) funded Human Health Exposure Analysis Resource Program (HHEAR): U2CES030857 (Du/Fennell/SS) and the Sumner lab. This work was also supported by the National Institute of Child Health and Human Development (R21HD087878) and the Bernick Faculty Grant program to EH.

## Conflict of interest

The authors declare that the research was conducted in the absence of any commercial or financial relationships that could be construed as a potential conflict of interest.

## Publisher’s note

All claims expressed in this article are solely those of the authors and do not necessarily represent those of their affiliated organizations, or those of the publisher, the editors and the reviewers. Any product that may be evaluated in this article, or claim that may be made by its manufacturer, is not guaranteed or endorsed by the publisher.

## Supplementary material

The Supplementary material for this article can be found online at: https://www.frontiersin.org/articles/10.3389/fnut.2023.1144131/full#supplementary-material
