# Supplementary material for: Untargeted metabolomics on first trimester serum implicates metabolic perturbations associated with BMI in development of hypertensive disorders: a discovery study
Source: Front Nutr. 2023 Jul 17;10:1144131. doi: 10.3389/fnut.2023.1144131 (PMC10388370; doi:10.3389/fnut.2023.1144131)
Supplement: Supplementary file 1 [file Data_Sheet_1.docx]

**SUPPLEMENTAL MATERIAL**

Untargeted Metabolomics on First Trimester Serum Implicates Metabolic Perturbations Associated with BMI as a factor in Pregnancy Hypertensive Disorders: A Discovery Study

K Pan,^1†^ Y-Y Li, ^2†^ S McRitchie,^2^ EW Harville^1†^, SJ. Sumner^2*^

1. Department of Epidemiology, Tulane University School of Public Health and Tropical Medicine, New Orleans, LA
2. Nutrition Research Institute, Department of Nutrition, University of North Carolina at Chapel Hill School of Public Health, Chapel Hill, NC 27599, USA

† Co-first authors

*Corresponding Author

Susan CJ Sumner, PhD

Professor, Department of Nutrition

Nutrition Research Institute

University of North Carolina at Chapel Hill

Chapel Hill, NC, 27514, USA

Tel: 919-622-4456

Email: Susan_sumner@unc.edu

* Corresponding Author

EW Harville, PhD

Tulane School of Public Health and Tropical Medicine

Epidemiology #8318

1440 Canal St. Ste. 2001

New Orleans, LA 70112

harville@tulane.edu

Tel. 504-988-7327

Fax. 504-988-1568

**Table S1. The BMI-associated metabolites in pregnant women with (n=49) and without HDP (n=105)**

| **Code** | **Peak/Signal** | **ID** | **Metabolites** | **Ontology** | **Without HDP** | | **With HDP** | |
| --- | --- | --- | --- | --- | --- | --- | --- | --- |
|  |  |  |  |  | **Difference *** | **FDR value^#^** | **Difference*** | **FDR value^#^** |
| A | 11.10_543.2801m/z | HMDB0010320 | Cortolone-3-glucuronide | PDd | 3.76 | 0.001 | 5.38 | 0.031 |
| A | 13.56_402.3130n | 73560 | Androsterone Enanthate | PDd | 3.53 | 0.004 | 6.61 | 0.001 |
| A | 13.10_605.2634m/z | N/A | N/A | N/A | 2.75 | 0.016 | 5.73 | 0.021 |
| B | 5.02_230.1024m/z | 160637 | 3-(3-Methoxy-4hydroxyphenyl)lactate | OL_2a | 2.40 | 0.043 |  |  |
| B | 3.84_132.0809m/z | 91462 | Nornicotine | OL_2b | 2.41 | 0.038 |  |  |
| B | 11.36_299.2368m/z | 1605-89-6 | Bolasterone | Pda | -2.93 | 0.013 |  |  |
| B | 7.39_359.2286m/z | 22097 | Asn Ile Leu | Pda | 2.88 | 0.011 |  |  |
| B | 1.01_305.1518n | 23655 | Met Arg | Pda | 2.91 | 0.010 |  |  |
| B | 3.04_322.1872m/z | 23811 | Arg Phe | Pda | 2.60 | 0.022 |  |  |
| B | 2.10_274.1032m/z | HMDB0000052 | Argininosuccinic acid | PDb | 3.77 | 0.001 |  |  |
| B | 11.15_275.1641m/z | HMDB0040926 | 2-Butyl-5-[2-(4-hydroxy-3-methoxyphenyl)ethyl]furan | PDb | 2.57 | 0.025 |  |  |
| B | 2.92_304.1502m/z | HMDB0039423 | N5-Acetyl-N2-gamma-L-glutamyl-L-ornithine | PDb | 2.70 | 0.019 |  |  |
| B | 4.55_395.1925m/z | HMDB0041908 | Imidaprilat | PDb | 2.32 | 0.044 |  |  |
| B | 2.47_806.5132n | HMDB0060612 | N-di-Demethyl roxithromycin | PDb | 2.33 | 0.049 |  |  |
| B | 15.42_567.3313n | HMDB0010404 | LysoPC(22:6(4Z,7Z,10Z,13Z,16Z,19Z) | PDb | -2.83 | 0.025 |  |  |
| B | 14.07_351.2169n | HMDB0060061 | Sphingosine 1-phosphate (d16:1-P) | PDb | 2.32 | 0.050 |  |  |
| B | 4.70_261.0869m/z | HMDB0032102 | 1,2,3,4-Tetrahydro-b-carboline-1,3-dicarboxylic acid | PDc | 2.86 | 0.013 |  |  |
| B | 4.12_405.2344m/z | eew9ZJLg0hhPwvK | Glu-Pro-Lys | PDc | 2.94 | 0.011 |  |  |
| B | 0.51_275.1711m/z | HMDB0028949 | Lysyl-Glutamine | PDc | 2.57 | 0.032 |  |  |
| B | 2.52_175.0602m/z | HMDB0061388 | Dimethyl 2-oxoglutarate | PDc | -2.34 | 0.043 |  |  |
| B | 4.25_516.2781m/z | HMDB0014936 | Gentamicin | PDc | 3.01 | 0.010 |  |  |
| B | 14.56_383.2942m/z | HMDB0005814 | Testosterone enanthate | PDc | -2.41 | 0.037 |  |  |
| B | 1.01_257.0266m/z | HMDB0059808 | Acetyl citrate | PDc | 3.59 | 0.004 |  |  |
| B | 13.05_533.2719m/z | qY8D9Py7B4iFJXL | Tyr-Tyr-Arg | PDc | -2.51 | 0.040 |  |  |
| B | 11.36_533.2721m/z | 157283-68-6 | (+)-Fluprostenol isopropyl ester | PDc | -2.79 | 0.013 |  |  |
| B | 3.37_241.0930m/z | HMDB0060589 | Aminofurantoin | PDc | 3.09 | 0.007 |  |  |
| B | 4.35_125.0478n | 128-53-0 | N-Ethylmaleimide | PDc | -2.92 | 0.010 |  |  |
| B | 5.28_553.2858n | 264397 | Ile Glu Val His Gly | PDc | 2.67 | 0.020 |  |  |
| B | 0.61_289.1615m/z | HMDB0028725 | Asparaginyl-Arginine | PDc | 3.23 | 0.006 |  |  |
| B | 9.72_416.3521m/z | HMDB0034731 | Tomatidine | PDc | 2.51 | 0.030 |  |  |
| B | 2.25_192.0867m/z | HMDB0029433 | L-2-Amino-4-methylenepentanedioic acid | PDc | 3.06 | 0.015 |  |  |
| B | 4.70_375.0975m/z | HMDB0060617 | Penicilloic acid | PDd | 2.96 | 0.010 |  |  |
| B | 2.69_206.0481m/z | HMDB0034782 | Benzyl sulfate | PDd | -2.77 | 0.016 |  |  |
| B | 11.10_565.2617m/z | HMDB0010320 | Cortolone-3-glucuronide | PDd | 3.26 | 0.006 |  |  |
| B | 4.97_470.2127n | 157822 | His Ser Val Glu | PDd | 3.02 | 0.010 |  |  |
| B | 13.94_467.2646n | 64686 | 17-trifluoromethylphenyl trinor PGF2Î± ethyl amide | PDd | -3.15 | 0.007 |  |  |
| B | 9.34_234.0204n | HMDB0128030 | (4-ethyl-2,6-dihydroxyphenyl)oxidanesulfonic acid | PDd | -2.96 | 0.010 |  |  |
| B | 15.23_432.3234n | HMDB0012454 | 3beta,7alpha-Dihydroxy-5-cholestenoate | PDd | -3.77 | 0.003 |  |  |
| B | 7.01_500.2717m/z | j6R0FazdNLeMUcX | Arg-Phe-Arg | PDd | -2.39 | 0.043 |  |  |
| B | 3.41_165.5513m/z | N/A | N/A | N/A | -2.53 | 0.028 |  |  |
| B | 0.53_214.0804n | N/A | N/A | N/A | 2.36 | 0.043 |  |  |
| B | 3.61_209.1263m/z | N/A | N/A | N/A | 3.40 | 0.004 |  |  |
| B | 0.56_895.7887n | N/A | N/A | N/A | -2.83 | 0.016 |  |  |
| B | 7.79_691.3143m/z | N/A | N/A | N/A | 3.12 | 0.007 |  |  |
| B | 8.48_547.8199m/z | N/A | N/A | N/A | 3.39 | 0.004 |  |  |
| B | 7.24_530.0154m/z | N/A | N/A | N/A | 2.36 | 0.049 |  |  |
| B | 10.36_1198.6101n | N/A | N/A | N/A | 2.53 | 0.030 |  |  |
| B | 6.55_452.9051m/z | N/A | N/A | N/A | 2.90 | 0.010 |  |  |
| B | 9.09_1022.5013m/z | N/A | N/A | N/A | 3.12 | 0.009 |  |  |
| B | 5.43_356.1583m/z | N/A | N/A | N/A | 2.36 | 0.043 |  |  |
| B | 14.12_1015.9340m/z | N/A | N/A | N/A | -3.40 | 0.006 |  |  |
| B | 10.72_606.2846m/z | N/A | N/A | N/A | 3.04 | 0.015 |  |  |
| B | 13.23_517.2773m/z | N/A | N/A | N/A | -2.30 | 0.049 |  |  |
| B | 8.33_1032.6197m/z | N/A | N/A | N/A | 2.98 | 0.038 |  |  |
| B | 3.66_558.2309m/z | N/A | N/A | N/A | 3.06 | 0.009 |  |  |
| B | 6.50_585.6081m/z | N/A | N/A | N/A | 2.87 | 0.015 |  |  |
| B | 10.18_798.7560m/z | N/A | N/A | N/A | 2.79 | 0.017 |  |  |
| B | 14.10_1015.5807m/z | N/A | N/A | N/A | -2.52 | 0.043 |  |  |
| B | 12.59_896.6563m/z | N/A | N/A | N/A | 2.83 | 0.014 |  |  |
| B | 12.53_809.9179m/z | N/A | N/A | N/A | 2.76 | 0.020 |  |  |
| B | 13.99_427.2720n | N/A | N/A | N/A | -2.60 | 0.025 |  |  |
| B | 5.41_325.0923m/z | N/A | N/A | N/A | 2.50 | 0.031 |  |  |
| B | 3.39_276.6423m/z | N/A | N/A | N/A | 2.61 | 0.023 |  |  |
| B | 10.51_581.3172m/z | N/A | N/A | N/A | 2.80 | 0.016 |  |  |
| B | 7.79_683.3304m/z | N/A | N/A | N/A | 3.25 | 0.007 |  |  |
| B | 14.10_1015.8755m/z | N/A | N/A | N/A | -2.83 | 0.025 |  |  |
| B | 12.59_896.4892m/z | N/A | N/A | N/A | 2.79 | 0.017 |  |  |
| B | 9.14_695.0809m/z | N/A | N/A | N/A | 2.60 | 0.031 |  |  |
| B | 1.94_234.1529m/z | N/A | N/A | N/A | 2.45 | 0.038 |  |  |
| B | 14.10_982.7376n | N/A | N/A | N/A | -2.59 | 0.043 |  |  |
| B | 8.99_764.1749m/z | N/A | N/A | N/A | 2.80 | 0.013 |  |  |
| B | 14.12_1015.9932m/z | N/A | N/A | N/A | -3.09 | 0.010 |  |  |
| B | 8.99_841.1871m/z | N/A | N/A | N/A | 2.94 | 0.010 |  |  |
| B | 13.68_954.2641m/z | N/A | N/A | N/A | -2.85 | 0.042 |  |  |
| B | 7.64_808.8954m/z | N/A | N/A | N/A | 2.63 | 0.028 |  |  |
| B | 2.47_488.2465m/z | N/A | N/A | N/A | 3.05 | 0.008 |  |  |
| B | 3.92_237.6370m/z | N/A | N/A | N/A | 2.62 | 0.024 |  |  |
| B | 8.71_1012.3829m/z | N/A | N/A | N/A | 2.54 | 0.035 |  |  |
| B | 5.56_624.3228n | N/A | N/A | N/A | 2.92 | 0.013 |  |  |
| B | 0.61_254.0521n | N/A | N/A | N/A | 2.86 | 0.013 |  |  |
| B | 5.05_315.1661m/z | N/A | N/A | N/A | 2.83 | 0.013 |  |  |
| B | 11.71_1972.9283n | N/A | N/A | N/A | 2.72 | 0.017 |  |  |
| B | 5.89_603.2690m/z | N/A | N/A | N/A | 2.51 | 0.038 |  |  |
| B | 3.14_237.1576m/z | N/A | N/A | N/A | 3.03 | 0.009 |  |  |
| B | 12.48_1032.0594m/z | N/A | N/A | N/A | 2.62 | 0.028 |  |  |
| B | 13.68_954.0415m/z | N/A | N/A | N/A | -3.04 | 0.040 |  |  |
| B | 12.51_806.1761m/z | N/A | N/A | N/A | 2.93 | 0.012 |  |  |
| B | 5.18_573.7626m/z | N/A | N/A | N/A | 2.85 | 0.013 |  |  |
| B | 8.10_444.2817m/z | N/A | N/A | N/A | 3.16 | 0.006 |  |  |
| B | 7.51_1567.6216n | N/A | N/A | N/A | 2.74 | 0.027 |  |  |
| B | 15.44_311.6327m/z | N/A | N/A | N/A | -2.82 | 0.017 |  |  |
| B | 8.71_1016.7787m/z | N/A | N/A | N/A | 2.80 | 0.019 |  |  |
| B | 4.70_421.1030m/z | N/A | N/A | N/A | 3.17 | 0.007 |  |  |
| B | 13.68_965.1575m/z | N/A | N/A | N/A | -2.88 | 0.028 |  |  |
| B | 7.66_742.3858n | N/A | N/A | N/A | 3.57 | 0.004 |  |  |
| B | 12.87_551.3066n | N/A | N/A | N/A | -2.48 | 0.033 |  |  |
| B | 13.68_967.8226m/z | N/A | N/A | N/A | -2.56 | 0.042 |  |  |
| B | 7.64_809.1465m/z | N/A | N/A | N/A | 2.42 | 0.042 |  |  |
| B | 4.17_847.3949n | N/A | N/A | N/A | 2.58 | 0.025 |  |  |
| B | 8.99_763.5732m/z | N/A | N/A | N/A | 2.69 | 0.020 |  |  |
| B | 5.00_457.1579m/z | N/A | N/A | N/A | 3.30 | 0.005 |  |  |
| B | 8.74_816.4079m/z | N/A | N/A | N/A | 3.14 | 0.006 |  |  |
| B | 2.50_357.5173m/z | N/A | N/A | N/A | 2.48 | 0.049 |  |  |
| B | 9.16_838.1401m/z | N/A | N/A | N/A | 2.51 | 0.031 |  |  |
| B | 5.08_476.8618m/z | N/A | N/A | N/A | 2.67 | 0.020 |  |  |
| B | 1.89_168.0174n | N/A | N/A | N/A | -2.53 | 0.036 |  |  |
| B | 10.94_1012.1108m/z | N/A | N/A | N/A | 2.55 | 0.030 |  |  |
| B | 8.99_763.7738m/z | N/A | N/A | N/A | 2.95 | 0.010 |  |  |
| B | 1.16_692.0937m/z | N/A | N/A | N/A | 2.59 | 0.039 |  |  |
| B | 12.51_782.9283n | N/A | N/A | N/A | 2.90 | 0.013 |  |  |
| B | 3.87_469.1980m/z | N/A | N/A | N/A | 2.55 | 0.043 |  |  |
| B | 5.89_804.0222m/z | N/A | N/A | N/A | 3.00 | 0.010 |  |  |
| B | 2.77_294.6585m/z | N/A | N/A | N/A | 2.42 | 0.035 |  |  |
| B | 9.57_839.4082m/z | N/A | N/A | N/A | 2.38 | 0.045 |  |  |
| B | 4.97_255.0869m/z | N/A | N/A | N/A | 3.05 | 0.010 |  |  |
| B | 6.02_373.1744m/z | N/A | N/A | N/A | 2.79 | 0.021 |  |  |
| B | 10.03_781.7211m/z | N/A | N/A | N/A | 2.38 | 0.047 |  |  |
| B | 3.22_463.9687m/z | N/A | N/A | N/A | 2.35 | 0.042 |  |  |
| B | 5.66_434.9877m/z | N/A | N/A | N/A | 2.87 | 0.018 |  |  |
| B | 8.79_727.3867m/z | N/A | N/A | N/A | 2.30 | 0.049 |  |  |
| B | 7.29_683.0036m/z | N/A | N/A | N/A | 2.64 | 0.042 |  |  |
| B | 3.17_328.6534m/z | N/A | N/A | N/A | 2.97 | 0.010 |  |  |
| B | 12.51_796.6893m/z | N/A | N/A | N/A | 2.93 | 0.012 |  |  |
| B | 6.45_533.9498m/z | N/A | N/A | N/A | 2.64 | 0.020 |  |  |
| B | 11.74_1037.5543m/z | N/A | N/A | N/A | 2.40 | 0.047 |  |  |
| B | 11.23_943.2119m/z | N/A | N/A | N/A | 2.35 | 0.049 |  |  |
| B | 2.15_264.6241m/z | N/A | N/A | N/A | 3.11 | 0.008 |  |  |
| B | 6.68_493.7671m/z | N/A | N/A | N/A | 2.32 | 0.050 |  |  |
| B | 6.88_334.6897m/z | N/A | N/A | N/A | 2.92 | 0.010 |  |  |
| B | 3.87_468.9473m/z | N/A | N/A | N/A | 2.56 | 0.038 |  |  |
| B | 3.09_278.1478m/z | N/A | N/A | N/A | 2.88 | 0.010 |  |  |
| B | 9.54_1435.7190n | N/A | N/A | N/A | 4.30 | 0.001 |  |  |
| B | 8.99_763.9745m/z | N/A | N/A | N/A | 2.81 | 0.013 |  |  |
| B | 3.66_508.2234m/z | N/A | N/A | N/A | 3.16 | 0.010 |  |  |
| B | 3.22_463.7179m/z | N/A | N/A | N/A | 2.56 | 0.025 |  |  |
| B | 10.84_862.9517m/z | N/A | N/A | N/A | 2.54 | 0.034 |  |  |
| B | 10.21_1285.6423n | N/A | N/A | N/A | 3.13 | 0.009 |  |  |
| B | 5.25_188.5445m/z | N/A | N/A | N/A | 3.04 | 0.009 |  |  |
| B | 9.90_932.1363m/z | N/A | N/A | N/A | 3.06 | 0.009 |  |  |
| B | 10.84_862.7845m/z | N/A | N/A | N/A | 2.55 | 0.031 |  |  |
| B | 5.41_611.2308m/z | N/A | N/A | N/A | 2.44 | 0.037 |  |  |
| B | 9.21_2058.9239n | N/A | N/A | N/A | 3.45 | 0.004 |  |  |
| B | 1.01_169.5948m/z | N/A | N/A | N/A | 2.72 | 0.019 |  |  |
| B | 4.30_573.2993m/z | N/A | N/A | N/A | 2.95 | 0.010 |  |  |
| B | 14.48_539.3586n | N/A | N/A | N/A | -3.08 | 0.009 |  |  |
| B | 10.72_600.9611m/z | N/A | N/A | N/A | 2.81 | 0.020 |  |  |
| B | 9.14_695.5827m/z | N/A | N/A | N/A | 2.68 | 0.025 |  |  |
| B | 5.28_556.2997m/z | N/A | N/A | N/A | 2.64 | 0.022 |  |  |
| B | 4.30_422.8618m/z | N/A | N/A | N/A | 2.83 | 0.017 |  |  |
| B | 0.64_513.9237m/z | N/A | N/A | N/A | -2.49 | 0.049 |  |  |
| B | 13.66_971.7151m/z | N/A | N/A | N/A | -2.59 | 0.040 |  |  |
| B | 4.25_277.6159m/z | N/A | N/A | N/A | 2.61 | 0.025 |  |  |
| B | 8.71_1009.3985n | N/A | N/A | N/A | 2.40 | 0.043 |  |  |
| B | 7.79_687.5726m/z | N/A | N/A | N/A | 2.77 | 0.020 |  |  |
| B | 10.08_698.2983m/z | N/A | N/A | N/A | 2.61 | 0.028 |  |  |
| B | 7.79_687.3218m/z | N/A | N/A | N/A | 3.13 | 0.008 |  |  |
| B | 9.16_837.8895m/z | N/A | N/A | N/A | 2.40 | 0.049 |  |  |
| B | 10.08_688.6321m/z | N/A | N/A | N/A | 2.54 | 0.036 |  |  |
| B | 8.58_1379.5840n | N/A | N/A | N/A | 3.64 | 0.003 |  |  |
| B | 13.68_965.4919m/z | N/A | N/A | N/A | -3.71 | 0.007 |  |  |
| B | 3.82_277.1344m/z | N/A | N/A | N/A | 6.95 | 0.007 |  |  |
| B | 2.87_245.6346m/z | N/A | N/A | N/A | 3.70 | 0.002 |  |  |
| B | 4.35_210.0074m/z | N/A | N/A | N/A | -2.68 | 0.023 |  |  |
| B | 5.59_873.4693n | N/A | N/A | N/A | 3.18 | 0.006 |  |  |
| B | 3.66_507.9727m/z | N/A | N/A | N/A | 3.05 | 0.010 |  |  |
| B | 11.23_1863.4306n | N/A | N/A | N/A | 2.52 | 0.030 |  |  |
| B | 5.48_405.2168m/z | N/A | N/A | N/A | 3.29 | 0.004 |  |  |
| B | 10.84_739.9603m/z | N/A | N/A | N/A | 2.99 | 0.010 |  |  |
| B | 13.68_965.2691m/z | N/A | N/A | N/A | -3.93 | 0.007 |  |  |
| B | 15.42_569.3382n | N/A | N/A | N/A | -2.52 | 0.043 |  |  |
| B | 13.68_965.3806m/z | N/A | N/A | N/A | -3.69 | 0.007 |  |  |
| B | 3.71_456.1969n | N/A | N/A | N/A | 2.52 | 0.030 |  |  |
| B | 14.10_959.4376m/z | N/A | N/A | N/A | -2.59 | 0.043 |  |  |
| B | 6.57_330.6766m/z | N/A | N/A | N/A | 2.62 | 0.025 |  |  |
| B | 8.33_1205.5224n | N/A | N/A | N/A | 2.71 | 0.019 |  |  |
| B | 12.82_1013.5011m/z | N/A | N/A | N/A | 2.93 | 0.013 |  |  |
| B | 7.01_464.2218m/z | N/A | N/A | N/A | 2.54 | 0.028 |  |  |
| B | 1.94_208.6344m/z | N/A | N/A | N/A | 2.86 | 0.010 |  |  |
| B | 4.25_594.8780m/z | N/A | N/A | N/A | 2.90 | 0.017 |  |  |
| B | 9.21_2042.9545n | N/A | N/A | N/A | 3.07 | 0.010 |  |  |
| B | 5.28_296.6236m/z | N/A | N/A | N/A | 2.72 | 0.017 |  |  |
| B | 3.71_248.0791m/z | N/A | N/A | N/A | 2.57 | 0.026 |  |  |
| B | 1.32_264.1321m/z | N/A | N/A | N/A | 2.66 | 0.030 |  |  |
| B | 7.08_522.2842m/z | N/A | N/A | N/A | 2.87 | 0.013 |  |  |
| B | 10.03_2282.2128n | N/A | N/A | N/A | 3.09 | 0.010 |  |  |
| B | 5.33_474.7193m/z | N/A | N/A | N/A | 2.97 | 0.010 |  |  |
| B | 7.66_418.7474m/z | N/A | N/A | N/A | 2.38 | 0.042 |  |  |
| B | 15.46_445.2938n | N/A | N/A | N/A | -2.35 | 0.042 |  |  |
| B | 8.74_2386.2736n | N/A | N/A | N/A | 2.97 | 0.009 |  |  |
| B | 5.64_1097.4906n | N/A | N/A | N/A | 2.53 | 0.030 |  |  |
| B | 6.12_269.6547m/z | N/A | N/A | N/A | 2.78 | 0.013 |  |  |
| B | 4.92_825.4004n | N/A | N/A | N/A | 2.38 | 0.043 |  |  |
| B | 7.47_401.7321m/z | N/A | N/A | N/A | 2.42 | 0.038 |  |  |
| B | 5.10_1345.6528n | N/A | N/A | N/A | 2.64 | 0.021 |  |  |
| B | 7.01_866.4999n | N/A | N/A | N/A | 2.66 | 0.019 |  |  |
| B | 10.61_1948.0532n | N/A | N/A | N/A | 2.76 | 0.017 |  |  |
| B | 6.83_752.4565n | N/A | N/A | N/A | 2.48 | 0.031 |  |  |
| B | 10.08_2025.9269n | N/A | N/A | N/A | 2.61 | 0.029 |  |  |
| B | 7.56_534.3143m/z | N/A | N/A | N/A | 2.74 | 0.016 |  |  |
| B | 1.05_198.0286m/z | N/A | N/A | N/A | 2.67 | 0.026 |  |  |
| B | 6.57_365.7264m/z | N/A | N/A | N/A | 2.44 | 0.043 |  |  |
| B | 3.79_344.1927m/z | N/A | N/A | N/A | 2.92 | 0.010 |  |  |
| B | 1.92_243.0112m/z | N/A | N/A | N/A | -2.40 | 0.047 |  |  |
| B | 6.32_708.4153n | N/A | N/A | N/A | 3.51 | 0.004 |  |  |
| B | 6.17_417.2344m/z | N/A | N/A | N/A | 2.82 | 0.013 |  |  |
| B | 10.08_695.9595m/z | N/A | N/A | N/A | 2.85 | 0.014 |  |  |
| B | 7.79_691.5651m/z | N/A | N/A | N/A | 3.36 | 0.007 |  |  |
| B | 0.53_441.0318m/z | N/A | N/A | N/A | 2.50 | 0.031 |  |  |
| B | 9.26_392.1858m/z | N/A | N/A | N/A | -2.48 | 0.042 |  |  |
| B | 6.86_581.3408m/z | N/A | N/A | N/A | 2.94 | 0.010 |  |  |
| B | 11.23_949.2086m/z | N/A | N/A | N/A | 2.60 | 0.026 |  |  |
| B | 6.30_477.7721m/z | N/A | N/A | N/A | 2.50 | 0.030 |  |  |
| B | 10.84_862.6173m/z | N/A | N/A | N/A | 2.51 | 0.038 |  |  |
| B | 10.61_675.6580m/z | N/A | N/A | N/A | 3.20 | 0.007 |  |  |
| B | 3.76_349.6713m/z | N/A | N/A | N/A | 2.50 | 0.030 |  |  |
| B | 1.16_1680.2814n | N/A | N/A | N/A | 2.35 | 0.043 |  |  |
| B | 9.93_825.6692m/z | N/A | N/A | N/A | 2.49 | 0.032 |  |  |
| B | 5.20_400.7348m/z | N/A | N/A | N/A | 2.76 | 0.017 |  |  |
| B | 0.56_827.8016n | N/A | N/A | N/A | -2.46 | 0.042 |  |  |
| B | 12.82_1023.2383m/z | N/A | N/A | N/A | 2.66 | 0.026 |  |  |
| B | 9.41_830.6676m/z | N/A | N/A | N/A | 3.31 | 0.006 |  |  |
| B | 0.53_152.0250n | N/A | N/A | N/A | 2.50 | 0.028 |  |  |
| B | 14.10_1016.0520m/z | N/A | N/A | N/A | -2.82 | 0.020 |  |  |
| B | 8.71_1005.7951m/z | N/A | N/A | N/A | 2.38 | 0.042 |  |  |
| B | 5.18_808.3885n | N/A | N/A | N/A | 2.47 | 0.032 |  |  |
| B | 8.48_548.0706m/z | N/A | N/A | N/A | 3.36 | 0.005 |  |  |
| B | 11.89_976.0109m/z | N/A | N/A | N/A | 2.45 | 0.043 |  |  |
| B | 12.51_806.4269m/z | N/A | N/A | N/A | 2.87 | 0.013 |  |  |
| B | 5.59_889.4338n | N/A | N/A | N/A | 3.18 | 0.006 |  |  |
| B | 6.57_307.6683m/z | N/A | N/A | N/A | 2.45 | 0.031 |  |  |
| B | 5.66_435.2386m/z | N/A | N/A | N/A | 2.53 | 0.040 |  |  |
| B | 4.92_351.5246m/z | N/A | N/A | N/A | 3.19 | 0.006 |  |  |
| B | 8.02_637.2826m/z | N/A | N/A | N/A | 2.36 | 0.044 |  |  |
| B | 10.84_1034.9388n | N/A | N/A | N/A | 2.44 | 0.037 |  |  |
| B | 7.79_683.5812m/z | N/A | N/A | N/A | 2.55 | 0.031 |  |  |
| B | 9.77_1868.0270n | N/A | N/A | N/A | 2.41 | 0.045 |  |  |
| B | 7.64_2049.0704n | N/A | N/A | N/A | 2.57 | 0.030 |  |  |
| B | 14.10_982.6820n | N/A | N/A | N/A | -2.93 | 0.020 |  |  |
| B | 3.47_465.2222n | N/A | N/A | N/A | 3.18 | 0.006 |  |  |
| B | 13.68_954.1524m/z | N/A | N/A | N/A | -3.75 | 0.004 |  |  |
| B | 0.53_509.0195m/z | N/A | N/A | N/A | 2.49 | 0.028 |  |  |
| C | 14.51_631.3452m/z | 1114770-15-8 | 1-(9Z-Octadecenoyl)-sn-glycero-3-phospho-(1'-myo-inositol) | PDc |  |  | 5.28 | 0.045 |
| C | 12.71_427.2456m/z | HMDB0000447 | 7a,12a-Dihydroxy-3-oxo-4-cholenoic acid | PDc |  |  | 5.38 | 0.031 |
| C | 13.56_561.3428m/z | HMDB0034706 | Cucurbitacin C | PDd |  |  | 5.83 | 0.021 |
| C | 14.15_397.3101m/z | 309762-85-4 | Cholest-4-en-26-oic acid, 3-oxo | PDd |  |  | 5.68 | 0.031 |
| C | 8.66_762.1452m/z | N/A | N/A | N/A |  |  | -5.51 | 0.045 |
| C | 12.59_607.3355n | N/A | N/A | N/A |  |  | 5.65 | 0.021 |
| C | 14.40_687.3557m/z | N/A | N/A | N/A |  |  | 5.60 | 0.021 |

Linear regression models adjusting for maternal age, whether they smoked more than 100 cigarettes in their lifetime, and gestational week when serum samples and BMI were acquired, were used to determine signals associated with BMI.

Region codes (e.g., A, B, and C) correspond to the Venn diagram in Figure 2a.

Peak/signal indicates untargeted metabolomics signals retained in the regression models and show significant association with the outcomes.

ID is the lab identifier used to indicate the metabolites in the in-house physical standard library or the identifier used in public databases.

Ontology: OL1, highly confident identification based on matching with In-house physical standard library (IPSL) via retention time (RT, with RT error≤|0.5|), exact mass (MS, with mass error<5ppm), and tandem mass similarity (MS/MS, with similarity≥30); OL2a, confident identification based on matching with IPSL via MS and RT; OL2b, annotation for the isomer or derivatives of the compound listed but not the compound itself, based on matching with IPSL via MS and MS/MS; PDa, annotation based on matching with public database via MS and experimental MS/MS (could be the listed compound, or the isomer or derivatives of the listed compound); PDb, athe nnotation based on matching with public database via MS and predict MS/MS; PDc, annotation for the listed compound based on matching with public database via MS and isotopic similarity or adducts; PDd annotation for a listed compound based on matching with public database via the MS; N/A, peaks was not identified or annotated.

Gray area, the signal/metabolites showed no significant association with the outcomes.

* Difference indicates the change for each 1 unite increases in BMI

# False Discovery Rate (FDR) corrected p-value

**Table S2. The HDP associated Metabolites in different BMI categories: normal weight (NW, n=45), overweight (OW, n=48), and obese (OB, n=61)**

| **Region code** | **Peak/Signal** | **ID** | **Metabolites** | **Ontology** | **NW** | | **OW** | | **OB** | |
| --- | --- | --- | --- | --- | --- | --- | --- | --- | --- | --- |
|  |  |  |  |  | **OR*** | **p value#** | **OR*** | **p value#** | **OR*** | **P value#** |
| C | 13.23_455.2249m/z | 106062 | Ala His Leu Asp | PDd | 0.216 | 0.043 |  |  | 3.003 | 0.025 |
| C | 1.89_249.0394m/z | HMDB0032956 | 2-O-p-Coumaroyltartronic acid | PDc | 0.030 | 0.039 |  |  | 1.885 | 0.049 |
| D | 5.86_313.1070m/z | HMDB0037315 | Cerasinone | PDb |  |  | 2.127 | 0.044 | 3.545 | 0.005 |
| D | 15.86_531.2960m/z | N/A | N/A | N/A |  |  | 3.093 | 0.017 | 0.371 | 0.011 |
| D | 5.86_198.1319m/z | N/A | N/A | N/A |  |  | 2.057 | 0.050 | 3.397 | 0.008 |
| D | 15.97_541.3860m/z | N/A | N/A | N/A |  |  | 0.379 | 0.037 | 0.472 | 0.026 |
| E | 5.00_191.0583n | 1826 | 5-HYDROXYINDOLEACETATE | OL_1* | 2.694 | 0.033 |  |  |  |  |
| E | 11.28_219.1743m/z | 88-26-6 | 2,6-Di-tert-butyl-4-hydroxymethylphenol | PDa | 7.974 | 0.043 |  |  |  |  |
| E | 2.32_279.1008m/z | HMDB0034367 | gamma-Glutamylmethionine | PDb | 2.546 | 0.037 |  |  |  |  |
| E | 6.52_247.0158n | HMDB0062547 | 2-Hydroxyacetaminophen sulfate | PDb | 2.340 | 0.037 |  |  |  |  |
| E | 11.07_159.0806m/z | 88779 | 2-Methoxyphthalene | PDc | 3.172 | 0.049 |  |  |  |  |
| E | 13.84_541.2983m/z | 264061 | Thr Pro Pro Val Gln | PDc | 3.353 | 0.013 |  |  |  |  |
| E | 1.16_169.0493m/z | 287064 | methyl 3,4-dihydroxybenzoate | PDc | 0.312 | 0.048 |  |  |  |  |
| E | 4.35_125.0478n | 128-53-0 | N-Ethylmaleimide | PDc | 0.064 | 0.029 |  |  |  |  |
| E | 3.02_152.0568m/z | HMDB0000403 | 2-Hydroxyadenine | PDc | 2.900 | 0.041 |  |  |  |  |
| E | 15.72_127.1118m/z | HMDB0032451 | 4-Octen-3-one | PDc | 18.137 | 0.036 |  |  |  |  |
| E | 4.97_470.2127n | 157822 | His Ser Val Glu | PDd | 2.456 | 0.038 |  |  |  |  |
| E | 15.79_353.3048m/z | 14602-39-2 | 5(Z),8(Z),11(Z)-Eicosatrienoic acid methyl ester | PDd | 2.705 | 0.042 |  |  |  |  |
| E | 13.99_175.0755m/z | 57646-01-2 | 3,6-Dimethylchromone | PDd | 2.213 | 0.040 |  |  |  |  |
| E | 12.12_301.2160m/z | HMDB0010202 | 12-HEPE | PDd | 2.822 | 0.015 |  |  |  |  |
| E | 10.61_522.1792m/z | HMDB0060763 | 4-Hydroxy duloxetine glucuronide | PDd | 2.636 | 0.038 |  |  |  |  |
| E | 3.61_209.1263m/z | N/A | N/A | N/A | 2.571 | 0.020 |  |  |  |  |
| E | 13.68_657.3512n | N/A | N/A | N/A | 2.105 | 0.035 |  |  |  |  |
| E | 10.72_958.9218m/z | N/A | N/A | N/A | 3.072 | 0.028 |  |  |  |  |
| E | 0.56_90.9820m/z | N/A | N/A | N/A | 0.186 | 0.019 |  |  |  |  |
| E | 9.67_273.0165m/z | N/A | N/A | N/A | 9.282 | 0.006 |  |  |  |  |
| E | 10.72_959.1222m/z | N/A | N/A | N/A | 2.551 | 0.039 |  |  |  |  |
| E | 15.42_806.4609n | N/A | N/A | N/A | 0.179 | 0.043 |  |  |  |  |
| E | 9.09_1022.5013m/z | N/A | N/A | N/A | 2.132 | 0.048 |  |  |  |  |
| E | 0.59_913.4879n | N/A | N/A | N/A | 0.224 | 0.031 |  |  |  |  |
| E | 1.50_125.0297m/z | N/A | N/A | N/A | 0.039 | 0.035 |  |  |  |  |
| E | 3.92_364.8731m/z | N/A | N/A | N/A | 5.210 | 0.024 |  |  |  |  |
| E | 0.62_104.1004m/z | N/A | N/A | N/A | 2.507 | 0.042 |  |  |  |  |
| E | 0.59_618.6671n | N/A | N/A | N/A | 0.216 | 0.017 |  |  |  |  |
| E | 4.50_89.0386m/z | N/A | N/A | N/A | 4.557 | 0.023 |  |  |  |  |
| E | 8.99_841.1871m/z | N/A | N/A | N/A | 2.143 | 0.043 |  |  |  |  |
| E | 4.62_365.6900m/z | N/A | N/A | N/A | 2.816 | 0.049 |  |  |  |  |
| E | 8.99_436.0929m/z | N/A | N/A | N/A | 2.801 | 0.047 |  |  |  |  |
| E | 0.88_200.8769m/z | N/A | N/A | N/A | 0.128 | 0.033 |  |  |  |  |
| E | 14.64_659.3668n | N/A | N/A | N/A | 2.641 | 0.027 |  |  |  |  |
| E | 15.44_311.6327m/z | N/A | N/A | N/A | 3.115 | 0.046 |  |  |  |  |
| E | 8.71_1016.7787m/z | N/A | N/A | N/A | 2.898 | 0.018 |  |  |  |  |
| E | 10.56_158.0272m/z | N/A | N/A | N/A | 13.258 | 0.023 |  |  |  |  |
| E | 16.00_235.1690m/z | N/A | N/A | N/A | 3.081 | 0.050 |  |  |  |  |
| E | 15.13_258.6503m/z | N/A | N/A | N/A | 3.012 | 0.048 |  |  |  |  |
| E | 15.11_333.2120m/z | N/A | N/A | N/A | 0.003 | 0.033 |  |  |  |  |
| E | 1.03_232.0471m/z | N/A | N/A | N/A | 0.162 | 0.031 |  |  |  |  |
| E | 2.52_176.9846m/z | N/A | N/A | N/A | 2.664 | 0.038 |  |  |  |  |
| E | 6.63_244.1373m/z | N/A | N/A | N/A | 3.161 | 0.039 |  |  |  |  |
| E | 5.94_230.0428n | N/A | N/A | N/A | 3.052 | 0.027 |  |  |  |  |
| E | 0.54_623.8787m/z | N/A | N/A | N/A | 0.166 | 0.033 |  |  |  |  |
| E | 1.16_317.0390m/z | N/A | N/A | N/A | 2.934 | 0.042 |  |  |  |  |
| E | 9.54_267.1168m/z | N/A | N/A | N/A | 0.000 | 0.041 |  |  |  |  |
| E | 15.68_1072.6172n | N/A | N/A | N/A | 3.782 | 0.034 |  |  |  |  |
| E | 7.41_386.1553n | N/A | N/A | N/A | 2.756 | 0.022 |  |  |  |  |
| E | 10.79_367.1872n | N/A | N/A | N/A | 3.807 | 0.047 |  |  |  |  |
| E | 15.28_295.6584m/z | N/A | N/A | N/A | 3.837 | 0.030 |  |  |  |  |
| E | 5.99_275.0479m/z | N/A | N/A | N/A | 3.356 | 0.016 |  |  |  |  |
| E | 0.54_589.8853m/z | N/A | N/A | N/A | 0.244 | 0.045 |  |  |  |  |
| E | 6.52_147.0638m/z | N/A | N/A | N/A | 2.376 | 0.037 |  |  |  |  |
| E | 11.53_369.2028n | N/A | N/A | N/A | 3.342 | 0.036 |  |  |  |  |
| E | 1.85_133.0956m/z | N/A | N/A | N/A | 0.214 | 0.030 |  |  |  |  |
| E | 10.72_798.9348m/z | N/A | N/A | N/A | 3.124 | 0.032 |  |  |  |  |
| E | 13.03_675.3614n | N/A | N/A | N/A | 1.993 | 0.042 |  |  |  |  |
| E | 15.97_291.6842m/z | N/A | N/A | N/A | 3.264 | 0.043 |  |  |  |  |
| E | 1.08_189.9927m/z | N/A | N/A | N/A | 3.429 | 0.048 |  |  |  |  |
| E | 15.13_273.6276m/z | N/A | N/A | N/A | 2.724 | 0.041 |  |  |  |  |
| E | 0.59_690.6193m/z | N/A | N/A | N/A | 0.164 | 0.033 |  |  |  |  |
| E | 14.15_405.2248m/z | N/A | N/A | N/A | 3.126 | 0.030 |  |  |  |  |
| E | 6.40_1013.0246m/z | N/A | N/A | N/A | 2.596 | 0.039 |  |  |  |  |
| E | 10.72_958.5206m/z | N/A | N/A | N/A | 2.629 | 0.034 |  |  |  |  |
| E | 15.93_276.6790m/z | N/A | N/A | N/A | 2.757 | 0.031 |  |  |  |  |
| E | 10.72_958.7213m/z | N/A | N/A | N/A | 2.837 | 0.029 |  |  |  |  |
| F | 7.89_277.1182m/z | p8HuIU9LQr@1JEG | PyroGlu-Phe | Pda |  |  | 2.225 | 0.026 |  |  |
| F | 15.06_475.2695n | HMDB0011509 | LysoPE(18:3(9Z,12Z,15Z)/0:0) | PDb |  |  | 3.174 | 0.008 |  |  |
| F | 15.86_479.3007n | HMDB0011505 | LysoPE(18:1(11Z)/0:0) | PDb |  |  | 2.376 | 0.027 |  |  |
| F | 9.34_299.1389m/z | HMDB0037499 | 3'-N'-Acetylfusarochromanone | PDb |  |  | 0.130 | 0.045 |  |  |
| F | 0.73_161.0921m/z | 3613685 | ETHYL 3-UREIDOPROPIOTE | PDc |  |  | 3.138 | 0.013 |  |  |
| F | 2.52_137.0210m/z | HMDB0060461 | cis-Acetylacrylate | PDc |  |  | 0.365 | 0.035 |  |  |
| F | 7.16_208.1212n | 92-13-7 | Pilocarpine | PDc |  |  | 1.959 | 0.048 |  |  |
| F | 11.76_243.1592m/z | HMDB0030987 | 2-Carboxy-4-dodecanolide | PDc |  |  | 3.319 | 0.040 |  |  |
| F | 13.84_495.2565m/z | HMDB0014801 | Flumethasone Pivalate | PDd |  |  | 2.399 | 0.020 |  |  |
| F | 11.20_280.0966m/z | HMDB0033480 | Graveoline | PDd |  |  | 2.979 | 0.027 |  |  |
| F | 8.79_239.0891m/z | 114212-45-2 | 3-Oxo-1,8-octanedicarboxylic acid | PDd |  |  | 0.305 | 0.041 |  |  |
| F | 7.21_327.1227m/z | 720674-23-7 | 3-(3,4-Dimethoxyphenyl)-7-methoxy-4-methylcoumarin | PDd |  |  | 2.095 | 0.044 |  |  |
| F | 8.56_860.4289m/z | N/A | N/A | N/A |  |  | 0.450 | 0.048 |  |  |
| F | 1.55_168.9907m/z | N/A | N/A | N/A |  |  | 0.423 | 0.031 |  |  |
| F | 13.00_525.3301m/z | N/A | N/A | N/A |  |  | 0.080 | 0.031 |  |  |
| F | 3.12_457.7037m/z | N/A | N/A | N/A |  |  | 0.253 | 0.014 |  |  |
| F | 2.57_152.0646m/z | N/A | N/A | N/A |  |  | 0.431 | 0.043 |  |  |
| F | 8.56_861.0300m/z | N/A | N/A | N/A |  |  | 0.310 | 0.014 |  |  |
| F | 5.08_685.2673m/z | N/A | N/A | N/A |  |  | 0.346 | 0.042 |  |  |
| F | 2.20_142.9467m/z | N/A | N/A | N/A |  |  | 0.447 | 0.043 |  |  |
| F | 15.62_594.3432m/z | N/A | N/A | N/A |  |  | 0.260 | 0.023 |  |  |
| F | 5.08_362.1108m/z | N/A | N/A | N/A |  |  | 0.240 | 0.020 |  |  |
| F | 5.92_243.1203m/z | N/A | N/A | N/A |  |  | 2.341 | 0.037 |  |  |
| F | 14.74_254.1518n | N/A | N/A | N/A |  |  | 2.260 | 0.043 |  |  |
| F | 15.46_538.2752m/z | N/A | N/A | N/A |  |  | 2.035 | 0.047 |  |  |
| F | 5.56_447.0849m/z | N/A | N/A | N/A |  |  | 0.388 | 0.027 |  |  |
| F | 6.32_1183.6968n | N/A | N/A | N/A |  |  | 0.345 | 0.037 |  |  |
| F | 14.10_959.3820m/z | N/A | N/A | N/A |  |  | 0.333 | 0.035 |  |  |
| F | 9.85_478.5506m/z | N/A | N/A | N/A |  |  | 2.188 | 0.047 |  |  |
| F | 8.53_861.2295m/z | N/A | N/A | N/A |  |  | 0.446 | 0.042 |  |  |
| F | 8.84_632.3280m/z | N/A | N/A | N/A |  |  | 0.220 | 0.029 |  |  |
| F | 14.10_507.2291n | N/A | N/A | N/A |  |  | 0.248 | 0.031 |  |  |
| F | 4.30_181.0569m/z | N/A | N/A | N/A |  |  | 2.687 | 0.026 |  |  |
| F | 2.27_232.1510m/z | N/A | N/A | N/A |  |  | 2.032 | 0.042 |  |  |
| F | 3.34_134.9673m/z | N/A | N/A | N/A |  |  | 0.423 | 0.040 |  |  |
| F | 5.10_397.1955m/z | N/A | N/A | N/A |  |  | 0.199 | 0.032 |  |  |
| G | 0.94_244.0926m/z | 6175 | Cytidine | OL_1 |  |  |  |  | 1.964 | 0.027 |
| G | 8.89_197.0806m/z | 6858 | 3,4,5-trimethoxybenzaldehyde | OL_1 |  |  |  |  | 1.950 | 0.043 |
| G | 2.94_230.1387m/z | 53481615 | Butenylcarnitine | OL_2a |  |  |  |  | 3.741 | 0.015 |
| G | 8.38_170.0601m/z | 92904 | Indolelactic acid | OL_2b |  |  |  |  | 0.234 | 0.023 |
| G | 6.68_219.1128m/z | 903 | N-ACETYLSEROTONIN | OL_2b* |  |  |  |  | 2.874 | 0.014 |
| G | 13.36_339.2680m/z | 5283906 | Dehydrolithocholic acid | OL_2b |  |  |  |  | 2.598 | 0.029 |
| G | 11.79_430.2950m/z | 23617285 | Glycylcholic acid | OL_2b |  |  |  |  | 2.438 | 0.018 |
| G | 7.87_151.0753m/z | 10296 | 2-Phenylpropiote | OL_2b |  |  |  |  | 2.039 | 0.049 |
| G | 6.50_191.0315m/z | 547 | 3,4-DIHYDROXYPHENYLACETATE | OL_2b |  |  |  |  | 2.168 | 0.047 |
| G | 11.61_287.2003m/z | 63-05-8 | 4-Androstene-3,17-dione | PDa |  |  |  |  | 2.054 | 0.037 |
| G | 10.59_271.2054m/z | 53-43-0 | 5-Androsten-3.beta.-ol-17-one | Pda |  |  |  |  | 2.672 | 0.018 |
| G | 13.13_286.2295n | 215 | Retinol / Retinol skeleton | Pda |  |  |  |  | 2.069 | 0.035 |
| G | 5.89_367.1275m/z | HMDB0029830 | N-Caffeoyltryptophan | PDb |  |  |  |  | 2.783 | 0.007 |
| G | 4.46_384.1147m/z | HMDB0000912 | Succinyladenosine | PDb |  |  |  |  | 2.611 | 0.005 |
| G | 15.04_379.2481n | HMDB0000277 | Sphingosine 1-phosphate | PDb |  |  |  |  | 0.490 | 0.022 |
| G | 4.80_335.1767n | HMDB0015549 | Hydroxychloroquine | PDb |  |  |  |  | 1.948 | 0.035 |
| G | 15.97_269.2473m/z | 10030-74-7 | Methyl hexadec-9-enoate | PDc |  |  |  |  | 0.505 | 0.030 |
| G | 15.97_351.2871m/z | HMDB0032477 | Polyoxyethylene 40 monostearate | PDc |  |  |  |  | 0.494 | 0.030 |
| G | 12.95_245.1148m/z | HMDB0038894 | Isoamyl p-anisate | PDc |  |  |  |  | 0.491 | 0.027 |
| G | 2.89_167.0703m/z | HMDB0125519 | 4-ethenyl-6-methoxybenzene-1,3-diol | PDc |  |  |  |  | 2.168 | 0.034 |
| G | 14.51_631.3452m/z | 1114770-15-8 | 1-(9Z-Octadecenoyl)-sn-glycero-3-phospho-(1'-myo-inositol) | PDc |  |  |  |  | 3.055 | 0.005 |
| G | 13.05_533.2719m/z | qY8D9Py7B4iFJXL | Tyr-Tyr-Arg | PDc |  |  |  |  | 1.899 | 0.035 |
| G | 14.23_445.3191n | HMDB0062337 | N-Oleoyl tyrosine | PDc |  |  |  |  | 4.091 | 0.017 |
| G | 7.56_199.1441m/z | HMDB0029140 | Valyl-Valine | PDc |  |  |  |  | 2.526 | 0.021 |
| G | 7.69_244.0848n | HMDB0006005 | Indolylacryloylglycine | PDc |  |  |  |  | 0.232 | 0.023 |
| G | 3.24_245.1495m/z | HMDB0028930 | Leucyl-Hydroxyproline | PDc |  |  |  |  | 2.249 | 0.044 |
| G | 8.99_226.0475m/z | 5460337Â Â | 3-(Indol-3-yl)pyruvate | PDc* |  |  |  |  | 0.092 | 0.038 |
| G | 8.91_242.0787m/z | 1037597-48-0 | Phosphoserine, butyl ester | PDc |  |  |  |  | 0.079 | 0.035 |
| G | 4.30_204.0511m/z | HMDB0028762 | Aspartyl-Serine | PDc |  |  |  |  | 2.337 | 0.041 |
| G | 3.02_195.0290m/z | HMDB0136660 | 4,7,8-trihydroxy-2H-chromen-2-one | PDd |  |  |  |  | 0.530 | 0.040 |
| G | 13.71_606.3418n | HMDB0010361 | (23S)-23,25-dihdroxy-24-oxovitamine D3 23-(beta-glucuronide) | PDd |  |  |  |  | 2.262 | 0.017 |
| G | 10.23_167.0947n | HMDB0039837 | 2-(2-Furanyl)-3-piperidinol | PDd |  |  |  |  | 5.741 | 0.013 |
| G | 13.56_561.3428m/z | HMDB0034706 | Cucurbitacin C | PDd |  |  |  |  | 2.069 | 0.021 |
| G | 10.72_208.1098n | HMDB0030197 | (Z)-3-Oxo-2-(2-pentenyl)-1-cyclopenteneacetic acid | PDd |  |  |  |  | 2.429 | 0.012 |
| G | 14.64_777.4023m/z | HMDB0034211 | Agavoside B | PDd |  |  |  |  | 2.717 | 0.013 |
| G | 1.89_366.0607m/z | HMDB0015206 | Fludarabine | PDd |  |  |  |  | 2.928 | 0.025 |
| G | 11.94_233.1509m/z | HMDB0031014 | Methyl (2E,6Z)-dodecadienoate | PDd |  |  |  |  | 0.462 | 0.023 |
| G | 13.34_590.3324m/z | HMDB0002579 | Glycochenodeoxycholic acid 3-glucuronide | PDd |  |  |  |  | 4.762 | 0.013 |
| G | 13.48_223.1669m/z | HMDB0000638 | Dodecanoic acid | PDd |  |  |  |  | 0.538 | 0.039 |
| G | 11.05_510.2522m/z | HMDB0002644 | N-[(3a,5b,7a,12a)-3,7-dihydroxy-24-oxo-12-(sulfooxy)cholan-24-yl]-Glycine | PDd |  |  |  |  | 2.099 | 0.026 |
| G | 13.56_402.3130n | 1.32E+08 | 25-hydroxyvitamin D3-26,23-lactol | PDd* |  |  |  |  | 2.274 | 0.015 |
| G | 13.94_467.2646n | 64686 | 17-trifluoromethylphenyl trinor PGF2ÃŽÂ± ethyl amide | PDd |  |  |  |  | 2.153 | 0.020 |
| G | 5.69_195.1129m/z | 92-43-3 | 1-Phenylpyrazolidin-3-one | PDd |  |  |  |  | 4.431 | 0.004 |
| G | 1.89_167.0466m/z | 135-19-3 | .beta.-phthol | PDd |  |  |  |  | 2.828 | 0.025 |
| G | 10.54_197.1172m/z | HMDB0135672 | 4-(3-hydroxybutyl)-2-methoxyphenol | PDd |  |  |  |  | 2.135 | 0.026 |
| G | 14.15_397.3101m/z | 309762-85-4 | Cholest-4-en-26-oic acid, 3-oxo | PDd |  |  |  |  | 2.178 | 0.027 |
| G | 8.63_386.2107m/z | 96593 | JWH 210 N-(4-hydroxypentyl) metabolite | PDd |  |  |  |  | 0.539 | 0.037 |
| G | 14.20_687.3556m/z |  |  | PDd |  |  |  |  | 3.443 | 0.034 |
| G | 0.77_261.1080m/z | HMDB0011161 | L-alpha-glutamyl-L-hydroxyproline | PDd |  |  |  |  | 2.174 | 0.031 |
| G | 0.65_162.1254m/z | N/A | N/A | N/A |  |  |  |  | 0.557 | 0.049 |
| G | 5.51_211.0278m/z | N/A | N/A | N/A |  |  |  |  | 0.343 | 0.010 |
| G | 8.91_606.5187m/z | N/A | N/A | N/A |  |  |  |  | 0.425 | 0.046 |
| G | 15.18_692.9586m/z | N/A | N/A | N/A |  |  |  |  | 2.178 | 0.016 |
| G | 0.75_232.0916m/z | N/A | N/A | N/A |  |  |  |  | 0.412 | 0.027 |
| G | 2.20_193.9708m/z | N/A | N/A | N/A |  |  |  |  | 0.331 | 0.006 |
| G | 6.17_650.3145m/z | N/A | N/A | N/A |  |  |  |  | 2.067 | 0.042 |
| G | 12.59_896.6563m/z | N/A | N/A | N/A |  |  |  |  | 0.519 | 0.039 |
| G | 13.26_695.3279n | N/A | N/A | N/A |  |  |  |  | 4.345 | 0.014 |
| G | 2.10_85.0478m/z | N/A | N/A | N/A |  |  |  |  | 2.295 | 0.020 |
| G | 7.97_372.1951m/z | N/A | N/A | N/A |  |  |  |  | 0.453 | 0.023 |
| G | 6.17_344.6342m/z | N/A | N/A | N/A |  |  |  |  | 2.081 | 0.032 |
| G | 1.89_127.0455m/z | N/A | N/A | N/A |  |  |  |  | 2.083 | 0.031 |
| G | 12.59_896.4892m/z | N/A | N/A | N/A |  |  |  |  | 0.544 | 0.046 |
| G | 14.43_416.1871m/z | N/A | N/A | N/A |  |  |  |  | 0.527 | 0.046 |
| G | 9.44_792.3963m/z | N/A | N/A | N/A |  |  |  |  | 0.538 | 0.037 |
| G | 1.03_188.0392m/z | N/A | N/A | N/A |  |  |  |  | 2.661 | 0.010 |
| G | 6.12_607.8045m/z | N/A | N/A | N/A |  |  |  |  | 0.417 | 0.027 |
| G | 14.79_536.3016m/z | N/A | N/A | N/A |  |  |  |  | 1.917 | 0.034 |
| G | 11.79_314.1493n | N/A | N/A | N/A |  |  |  |  | 2.322 | 0.034 |
| G | 0.56_1017.2949m/z | N/A | N/A | N/A |  |  |  |  | 0.486 | 0.026 |
| G | 4.48_651.2235m/z | N/A | N/A | N/A |  |  |  |  | 0.504 | 0.037 |
| G | 13.99_291.2012m/z | N/A | N/A | N/A |  |  |  |  | 0.432 | 0.024 |
| G | 13.74_338.1804m/z | N/A | N/A | N/A |  |  |  |  | 0.444 | 0.016 |
| G | 13.68_965.1575m/z | N/A | N/A | N/A |  |  |  |  | 1.855 | 0.042 |
| G | 13.68_967.9341m/z | N/A | N/A | N/A |  |  |  |  | 2.171 | 0.029 |
| G | 0.61_343.8616m/z | N/A | N/A | N/A |  |  |  |  | 0.532 | 0.046 |
| G | 5.38_254.0734m/z | N/A | N/A | N/A |  |  |  |  | 0.519 | 0.041 |
| G | 1.50_216.0311n | N/A | N/A | N/A |  |  |  |  | 3.562 | 0.036 |
| G | 0.99_187.0213m/z | N/A | N/A | N/A |  |  |  |  | 3.113 | 0.008 |
| G | 1.03_348.0696n | N/A | N/A | N/A |  |  |  |  | 1.922 | 0.031 |
| G | 14.72_675.3708m/z | N/A | N/A | N/A |  |  |  |  | 1.894 | 0.043 |
| G | 5.00_259.0488m/z | N/A | N/A | N/A |  |  |  |  | 3.128 | 0.006 |
| G | 3.66_508.2234m/z | N/A | N/A | N/A |  |  |  |  | 0.534 | 0.048 |
| G | 10.64_385.1833m/z | N/A | N/A | N/A |  |  |  |  | 0.461 | 0.022 |
| G | 4.78_213.1234m/z | N/A | N/A | N/A |  |  |  |  | 2.455 | 0.049 |
| G | 8.99_307.0500m/z | N/A | N/A | N/A |  |  |  |  | 0.048 | 0.010 |
| G | 8.48_487.6750m/z | N/A | N/A | N/A |  |  |  |  | 1.968 | 0.050 |
| G | 4.75_167.0580n | N/A | N/A | N/A |  |  |  |  | 3.905 | 0.021 |
| G | 10.31_159.1169m/z | N/A | N/A | N/A |  |  |  |  | 1.961 | 0.030 |
| G | 1.89_217.9874m/z | N/A | N/A | N/A |  |  |  |  | 2.221 | 0.030 |
| G | 1.87_357.0657m/z | N/A | N/A | N/A |  |  |  |  | 3.221 | 0.026 |
| G | 12.25_323.1464m/z | N/A | N/A | N/A |  |  |  |  | 0.526 | 0.045 |
| G | 12.33_447.2982n | N/A | N/A | N/A |  |  |  |  | 2.094 | 0.037 |
| G | 3.66_507.9727m/z | N/A | N/A | N/A |  |  |  |  | 0.542 | 0.047 |
| G | 5.92_243.1339m/z | N/A | N/A | N/A |  |  |  |  | 5.767 | 0.006 |
| G | 5.00_272.0207n | N/A | N/A | N/A |  |  |  |  | 8.320 | 0.003 |
| G | 13.68_965.2691m/z | N/A | N/A | N/A |  |  |  |  | 1.923 | 0.036 |
| G | 3.69_437.1843m/z | N/A | N/A | N/A |  |  |  |  | 0.399 | 0.036 |
| G | 4.30_298.0540m/z | N/A | N/A | N/A |  |  |  |  | 2.173 | 0.039 |
| G | 13.68_965.3806m/z | N/A | N/A | N/A |  |  |  |  | 1.877 | 0.045 |
| G | 11.61_503.2248m/z | N/A | N/A | N/A |  |  |  |  | 2.186 | 0.029 |
| G | 7.21_243.0208n | N/A | N/A | N/A |  |  |  |  | 0.475 | 0.026 |
| G | 8.53_1103.3850n | N/A | N/A | N/A |  |  |  |  | 2.010 | 0.036 |
| G | 1.50_260.0212n | N/A | N/A | N/A |  |  |  |  | 6.284 | 0.037 |
| G | 8.99_292.0503n | N/A | N/A | N/A |  |  |  |  | 0.031 | 0.007 |
| G | 6.78_575.9934m/z | N/A | N/A | N/A |  |  |  |  | 0.257 | 0.049 |
| G | 12.59_607.3355n | N/A | N/A | N/A |  |  |  |  | 5.030 | 0.009 |
| G | 8.68_901.3495n | N/A | N/A | N/A |  |  |  |  | 2.477 | 0.015 |
| G | 15.42_819.4655m/z | N/A | N/A | N/A |  |  |  |  | 1.854 | 0.044 |
| G | 3.12_475.2125m/z | N/A | N/A | N/A |  |  |  |  | 0.523 | 0.034 |
| G | 5.00_289.0003m/z | N/A | N/A | N/A |  |  |  |  | 2.776 | 0.020 |
| G | 3.74_387.7088m/z | N/A | N/A | N/A |  |  |  |  | 1.885 | 0.045 |
| G | 8.76_864.8187m/z | N/A | N/A | N/A |  |  |  |  | 0.501 | 0.028 |
| G | 8.74_689.3515m/z | N/A | N/A | N/A |  |  |  |  | 0.445 | 0.041 |
| G | 1.50_213.0048m/z | N/A | N/A | N/A |  |  |  |  | 2.274 | 0.029 |
| G | 1.87_271.0290m/z | N/A | N/A | N/A |  |  |  |  | 2.164 | 0.023 |
| G | 5.23_316.1756m/z | N/A | N/A | N/A |  |  |  |  | 1.988 | 0.033 |
| G | 5.20_322.0940n | N/A | N/A | N/A |  |  |  |  | 3.027 | 0.004 |
| G | 13.26_680.2707m/z | N/A | N/A | N/A |  |  |  |  | 4.438 | 0.017 |
| G | 9.54_440.3158m/z | N/A | N/A | N/A |  |  |  |  | 0.448 | 0.037 |
| G | 14.40_687.3557m/z | N/A | N/A | N/A |  |  |  |  | 6.904 | 0.002 |
| G | 5.13_509.2731m/z | N/A | N/A | N/A |  |  |  |  | 0.361 | 0.011 |
| G | 4.57_207.0930n | N/A | N/A | N/A |  |  |  |  | 2.579 | 0.035 |
| G | 12.87_304.2996m/z | N/A | N/A | N/A |  |  |  |  | 0.527 | 0.030 |
| G | 11.02_303.1517n | N/A | N/A | N/A |  |  |  |  | 2.667 | 0.005 |
| G | 8.76_847.1858n | N/A | N/A | N/A |  |  |  |  | 0.414 | 0.009 |
| G | 1.50_218.0258m/z | N/A | N/A | N/A |  |  |  |  | 5.140 | 0.035 |
| G | 4.35_398.6848m/z | N/A | N/A | N/A |  |  |  |  | 1.921 | 0.040 |
| G | 8.99_143.0731m/z | N/A | N/A | N/A |  |  |  |  | 0.071 | 0.013 |
| G | 1.89_189.5573m/z | N/A | N/A | N/A |  |  |  |  | 2.294 | 0.015 |
| G | 13.68_954.1524m/z | N/A | N/A | N/A |  |  |  |  | 2.160 | 0.028 |
| G | 1.16_459.0249m/z | N/A | N/A | N/A |  |  |  |  | 0.481 | 0.026 |

Logistic regression models adjusting for maternal age, whether they smoked more than 100 cigarettes in their lifetime, gestational week when serum samples and BMI were acquired, and gravidity (frequency-matching variable) were used to determine signals associated HDP in NW, OW, and OB, individually.

Region codes (e.g., C, D, E, F, G) correspond to the Venn diagram in Figure 3a.

Peak/signal indicates untargeted metabolomics signals retained in the regression models and show significant association with the outcomes.

ID is the lab identifier used to indicate the metabolites in the in-house physical standard library or the identifier used in public databases.

Ontology: OL1, highly confident identification based on matching with In-house physical standard library (IPSL) via retention time (RT, with RT error≤|0.5|), exact mass (MS, with mass error<5ppm), and tandem mass similarity (MS/MS, with similarity≥30); OL2a, confident identification based on matching with IPSL via MS and RT; OL2b, annotation for the isomer or derivatives of the compound listed but not the compound itself, based on matching with IPSL via MS and MS/MS; PDa, annotation based on matching with public database via MS and experimental MS/MS (could be the listed compound or the isomer or derivatives of the listed compound); PDb, athe nnotation based on matching with public database via MS and predict MS/MS; PDc, annotation for the listed compound based on matching with public database via MS and isotopic similarity or adducts; PDd annotation for a listed compound based on matching with public database via the MS; N/A, peaks was not identified or annotated.

Gray area, the signal/metabolites showed no significant association with the outcomes.

* OD, odds ratio indicates the level of association between HDP case and control

^#^ p-value indicates the significance of the association (p<0.05).

**FigureS1** **Box plots for metabolites in enriched Tryptophan pathway (Corresponding to figure 5 A and B in the maintext)**

1. Metabolites in tryptophan-microbial metablism


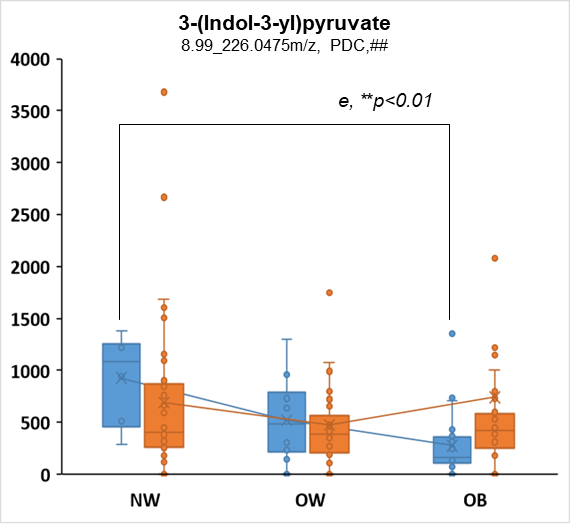

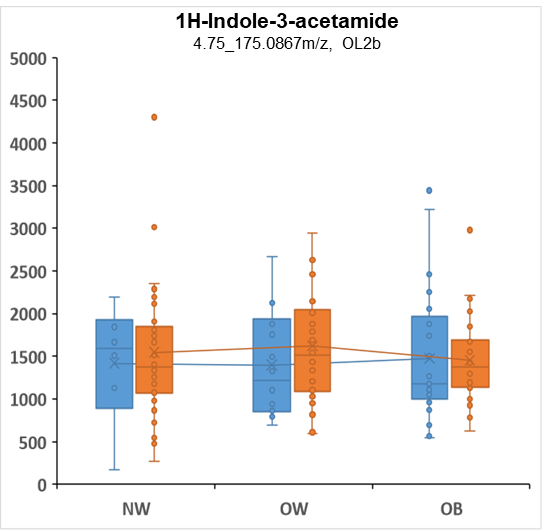

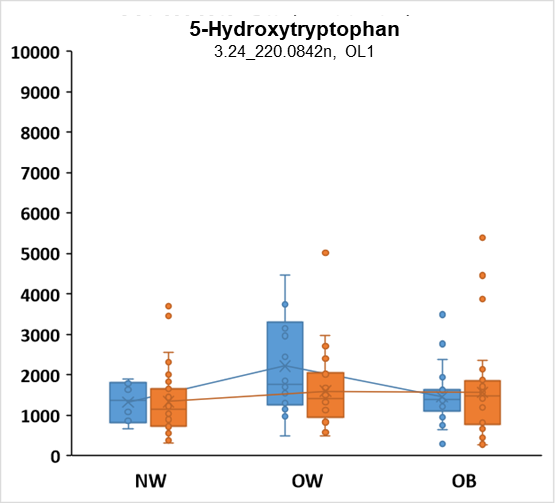

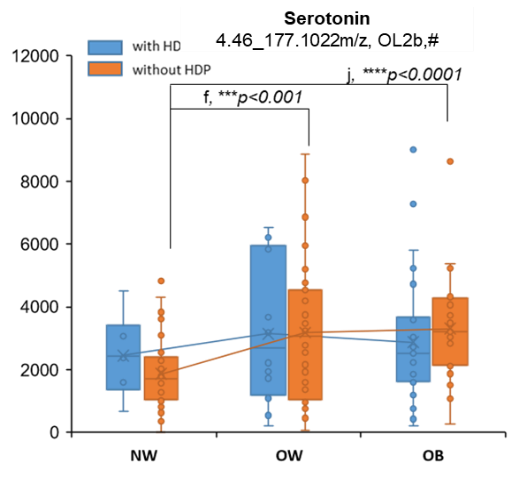

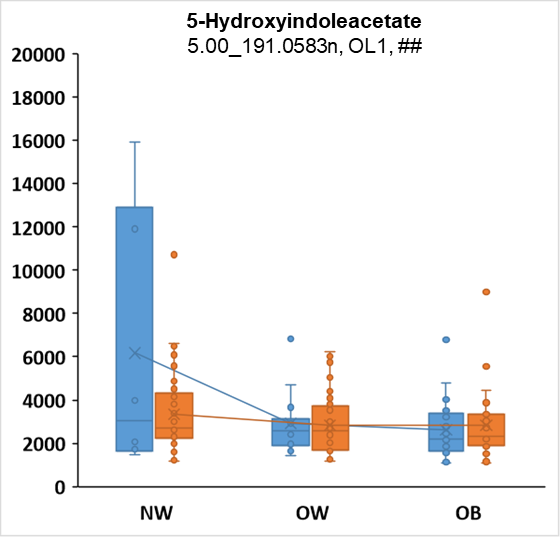

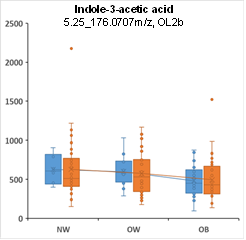

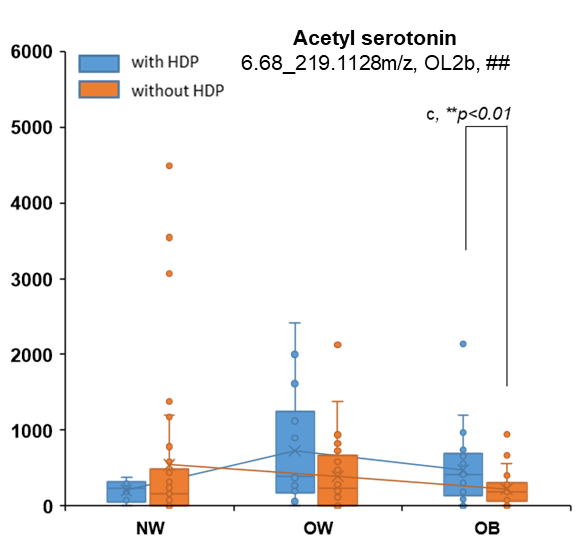

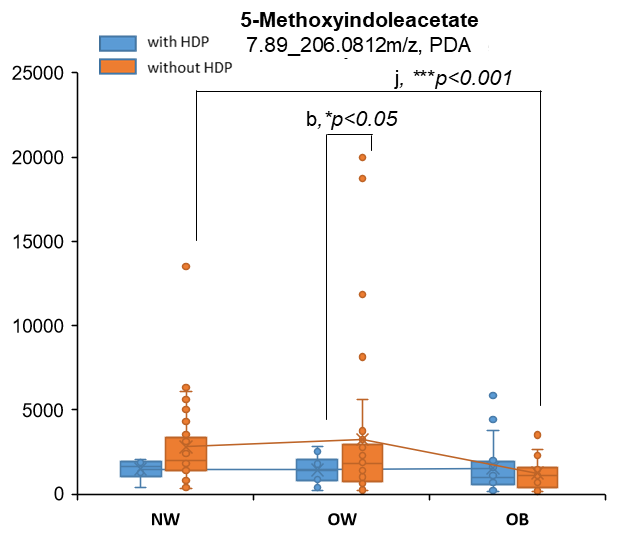

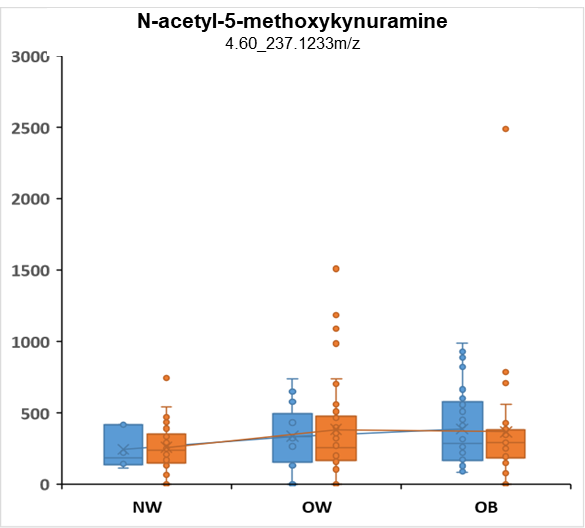


1. Metabolites in tryptophan-serotonine metablism
2.
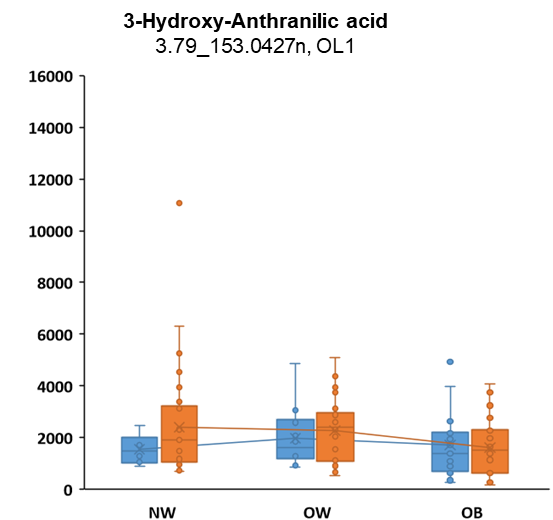

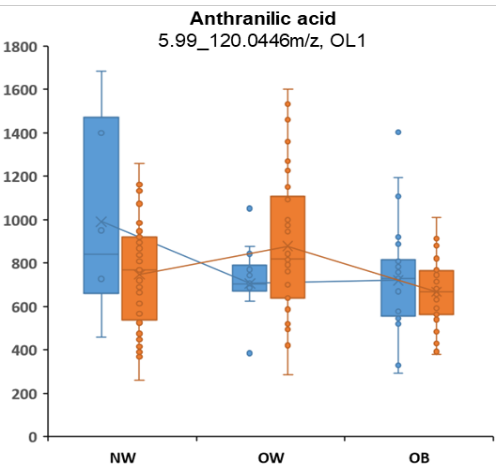

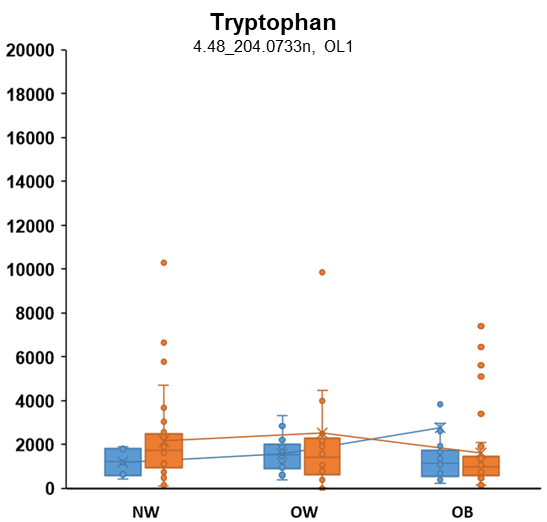
**Metabolites in tryptophan-** **Kynurenine pathway**

**
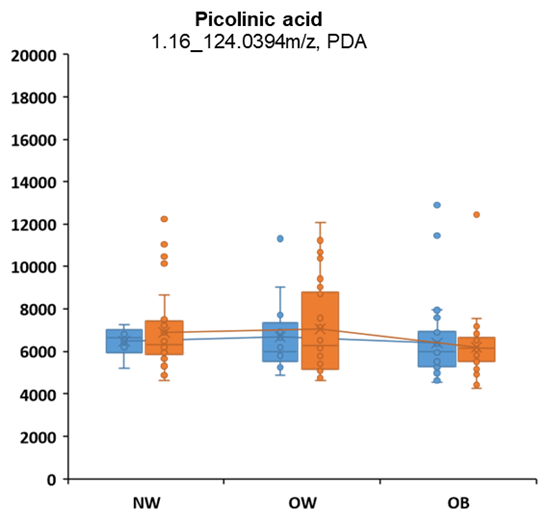

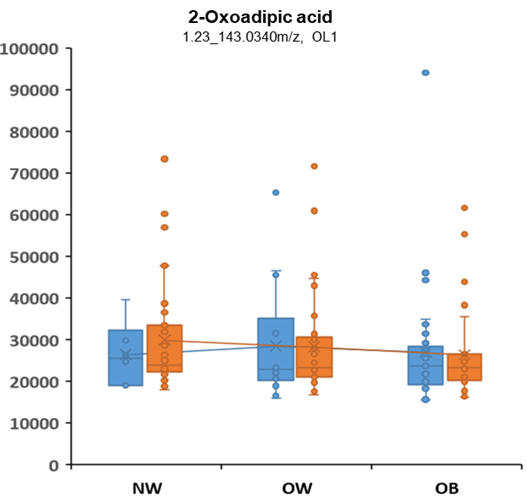
**

**Figure S1 Box plot of key metabolites in the enriched Tryptophan pathway**

The box plots of the identified/annotated metabolites were based on the relative peak intensity of the corresponding signal (RT_exact mass) and the classification with BMI categories (NW, normal weight; OW, overweight; OB, obese) and the HDP status (blue: with-HDP; orange: without HDP). The pairwise comparison was conducted by student t-test or Wilcoxon Rank-Sum Test (if the sample size in one of the groups is <10). Pairwise comparisons: a), with-HDP vs. without-HDP in the overweight women; b), with-HDP vs. without-HDP in the overweight women; c), with-HDP vs. without-HDP in the obese women; d) overweight vs. normal weight in women with HDP; e) obese vs. normal weight in women with HDP; f) overweight vs. normal weight in women without HDP; g) obese vs. normal weight in women without HDP. Metabolites with significant changes between the comparison groups with significant changes. *p<0.05,**p<0.01, ***p<0.001, and ****p<0.0001
